# The natural compound atraric acid suppresses androgen-regulated neo-angiogenesis of castration-resistant prostate cancer through angiopoietin 2

**DOI:** 10.1038/s41388-022-02333-7

**Published:** 2022-05-05

**Authors:** Marzieh Ehsani, Sophie Bartsch, Seyed Mohammad Mahdi Rasa, Jessica Dittmann, Thanakorn Pungsrinont, Laura Neubert, Soeren S. Huettner, Roland Kotolloshi, Katrin Schindler, Aya Ahmad, Alexander S. Mosig, Lisa Adam, Alessandro Ori, Francesco Neri, Alexander Berndt, Marc-Oliver Grimm, Aria Baniahmad

**Affiliations:** 1 Institute of Human Genetics, Jena University Hospital, Jena, 07740 Germany; 2 Leibniz Institute on Aging, Jena, 07745 Germany; 3 Institute of Biochemistry II, Jena University Hospital, Jena, 07740 Germany; 4 Institute of Pathology, Jena University Hospital, Jena, 07740 Germany; 5 Department of Adult and Pediatric Urology, Jena University Hospital, Jena, 07740 Germany

**Keywords:** Molecular biology, Prostate cancer, Endocrine cancer

## Abstract

Castration-resistant prostate cancer (CRPC) is an aggressive lethal form of prostate cancer (PCa). Atraric acid (AA) not only inhibits the wild-type androgen receptor (AR) but also those AR mutants that confer therapy resistance to other clinically used AR antagonists, indicating a different mode of AR antagonism. AA induces cellular senescence and inhibits CRPC tumour growth in in vivo xenograft mouse model associated with reduced neo-angiogenesis suggesting the repression of intratumoural neo-angiogenesis by AA. In line with this, the secretome of CRPC cells mediates neo-angiogenesis in an androgen-dependent manner, which is counteracted by AA. This was confirmed by two in vitro models using primary human endothelial cells. Transcriptome sequencing revealed upregulated angiogenic pathways by androgen, being however VEGF-independent, and pointing to the pro-angiogenic factor angiopoietin 2 (ANGPT2) as a key driver of neo-angiogenesis induced by androgens and repressed by AA. In agreement with this, AA treatment of native patient-derived PCa tumour samples ex vivo inhibits ANGPT2 expression. Mechanistically, in addition to AA, immune-depletion of ANGPT2 from secretome or blocking ANGPT2-receptors inhibits androgen-induced angiogenesis. Taken together, we reveal a VEGF-independent ANGPT2-mediated angiogenic pathway that is inhibited by AA leading to repression of androgen-regulated neo-angiogenesis.

## Introduction

Prostate cancer (PCa) is the most commonly diagnosed cancer and the second leading cause of cancer-related deaths in men in Western countries [1]. The growth of both the normal prostate and PCa is regulated by the androgen receptor (AR). Consistently, the AR is one of the main targets in hormonal therapy using androgen deprivation (ADT) and AR antagonists. PCa develops from a castration-sensitive tumour (CSPC) with or without metastasis into the more lethal castration-resistant PCa (CRPC) and metastatic CRPC (mCRPC). During this tumour evolution, not only a resistance to ADT but also resistance to other cancer inhibitory drugs can occur leading to drug resistant PCa (DRPC) [2]. The mechanisms of acquiring therapy resistance seem to be multifaceted and often multifactorial, including AR amplifications, AR mutants and AR variants in combination with changes of intracellular signal transduction and tumour environment [3].

The natural compound atraric acid (AA) was isolated from bark extracts of the African tree *Pygeum africanum*. Traditionally it has been used against prostate adenomas and was identified as an AR antagonist by our group [4]. AA is a non-steroidal compound with only one benzene ring that competes with androgens for binding to the AR, selectively inhibits AR-mediated transactivation, and is to our knowledge the first identified natural AR antagonist [5]. Mechanistically, AA decelerates the agonist-induced AR nuclear translocation and inhibits amino/carboxy-terminal interaction of AR and interaction with chromatin, leading to reduced DNA binding of the AR [6]. Further, AA inhibits androgen-dependent growth of CSPC cells and suppresses cell invasion through the extracellular matrix [5]. In previous studies we showed that AA induces cellular senescence in CSPC cells in vitro as well as ex vivo in patient PCa tumour samples [6].

Interestingly, in contrast to other second-generation AR antagonists, AA consists chemically of only one benzene ring thus being a smaller molecule. This raises the question whether AA also inhibits CRPC and whether it exhibits a distinct mechanism of action on AR compared to clinically used AR antagonists.

Cancer growth requires neo-angiogenesis for metastasis as well as growth of non-metastatic PCa. In general, neo-angiogenesis is controlled by a balance of secreted factors exhibiting pro- and anti-angiogenic activities. Although the vascular endothelial growth factor A (*VEGFA*) gene promoter contains an androgen response element, the link between AR signalling and angiogenesis in non-metastatic CRPC is largely unresolved [7]. Interestingly, an androgen regulation of angiogenesis has recently been described in non-prostate cells [7, 8] with a focus on VEGF. Despite high VEGFA levels in advanced PCa, anti-angiogenic therapies targeting VEGF or the VEGF receptor pathway have failed to provide significant treatment benefits in clinical trials [9, 10]. One underlying reason may be that PCa responds with an adaptive signalling and/or by other secreted pro-angiogenic factors that are distinct and independent from VEGF signalling.

Here, we show by using AR mutants, which mediate drug resistance, that AA possesses a distinct AR antagonism compared to other clinically used AR antagonists. Furthermore, we characterise AA as an inhibitor of CRPC tumour cell growth using the established human C4-2 cells as a CRPC model. Thereby, AA inhibits the neo-angiogenesis and growth of xenografted human CRPC tumours in vivo. The secretome of AR ligand-treated C4-2 cells indicates no regulation of VEGF by androgens and/or AA. However, the pro-angiogenic factor ANGPT2, is robustly induced and secreted by androgen-treated cells. This effect is counteracted by AA treatment. The androgen-induced secretome of CRPC cells enhanced tube formation and sprouting angiogenesis of spheroids generated by primary non-immortalised human endothelial cells. ANGPT2 is confirmed as a key factor to reduce neo-angiogenesis by both immune-depletion of ANGPT2 from the secretome and use of small molecule inhibitors targeting the ANGPT2 receptors. This reveals ANGPT2 as a major pro-angiogenic factor for a subset of PCa and as the major pro-angiogenic target of AA.

## Results

### Transcriptome analysis indicates AA-mediated inhibition of cell proliferation and induction of cellular senescence in CRPC cells

**Fig. 1:**
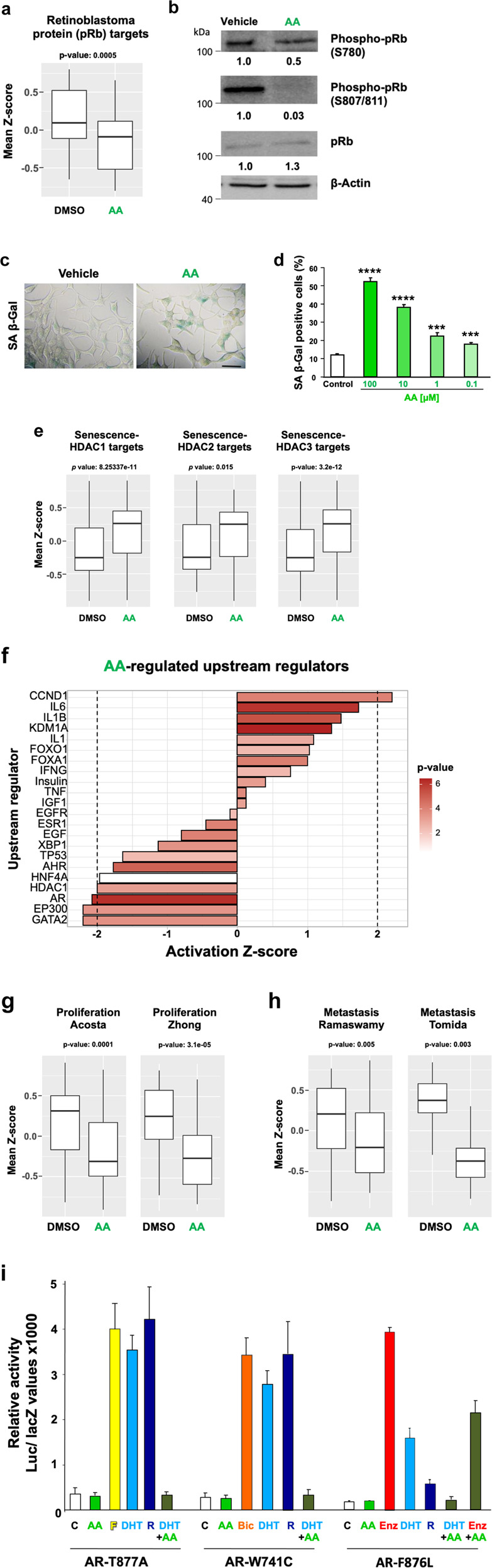
AA downregulates pRb-regulated genes and upregulates HDAC pathways of cellular senescence. Transcriptome sequencing was performed with the castration-resistant C4-2 cells treated with AA. **a** The retinoblastoma tumour suppressor, pRb, is a transcriptional repressor. GO gene sets identified from RNA-seq analysis of treated C4-2 cells with AA indicates that AA downregulates significantly pRb-regulated genes. **b** Western blot experiments reveal hypophosphorylation of pRb at serines 780 and 807/811 by AA. β-Actin was used as loading control. **c** Treatment with AA induces cellular senescence indicated by SA β-Gal activity staining. **d** AA induces cellular senescence in a concentration-dependent manner. **e** GO gene set analysis indicates HDAC1, 2 and 3 pathways are significantly induced by AA. **f** Activity prediction (by IPA software) of upstream regulators upon AA treatment compared to control. **g** RNA-seq analysis of GO gene sets suggests that AA treatment of C4-2 cells inhibits cell proliferation and **h** tumour metastasis pathways. For all the comparisons, Wilcoxon signed-rank test 2-sided was used. **i** AA reduces the androgen-mediated transactivation of AR mutants that are resistance to antagonist therapy. Expression vectors for mutant ARs AR-T877A, AR-W741C, and AR-F876L that mediate therapy resistance to clinically used AR antagonists, Flutamide (F), Bicalutamide (Bic) and Enzalutamide (Enz), respectively, were analysed in reporter assays. CV1 cells lacking AR were treated with the AR antagonists F, Bic, Enz (each 10 µM), AA (100 µM) and androgen DHT (10 nM), or (R1881 1 nM) in charcoal-treated 5% FBS. Results represent luciferase activity normalised to β-galactosidase activity derived from the co-transfected pCMV-lacZ vector. Error bars show standard deviation of the mean (SD). These results were verified by at least two additional replicates.

Transcriptome analysis was performed with the CRPC C4-2 cell line treated with AA or solvent control (DMSO). The gene ontology (GO) gene sets indicate that AA regulates the retinoblastoma protein (pRb) pathway (Fig. 1a). AA treatment leads to significant downregulation of pRb-regulated genes, indicating that pRb activity is enhanced by AA. pRb activity is regulated at its phosphorylation level. Thereby, hypohosphorylated pRb is a potent tumour suppressor and a key factor in PCa to inhibit cell proliferation [11, 12]. To confirm that AA regulates pRb phosphorylation levels, Western blotting experiments were performed with protein extracts of treated C4-2 cells. Treatment with AA reduced phosphorylation of pRb at serine 780 (S780) as well as serine 807 and 811 (S807/811; Fig. 1b). In androgen-dependent CSPC cells, the AR-pRb pathway was shown to regulate cellular senescence [6]. Accordingly, AA treatment induces the cell senescence marker senescence-associated β-galactosidase (SA β-Gal) activity in a dose-dependent manner (Fig. 1c, d) indicating that AA induces cellular senescence in the CRPC C4-2 cells. Upon knockdown of HDAC1, 2 and 3 cellular senescence can be induced [13], indicating that HDACs repress senescence-inducing genes. Performing gene set enrichment analysis (GSEA) indicates a significant induction of senescent related genes negatively regulated by HDAC1, HDAC 2 and HDAC 3 upon AA treatment (Fig. 1e). In agreement with GSEA analysis, IPA software also predicted besides AR itself also HDAC1 as one of the top upstream regulators inhibited by AA (Fig. 1f). In line with this notion, GSEA analysis also indicates a significant reduction of cell proliferation as well as metastatic potential (Fig. 1g, h). Thus, the transcriptome data indicate that AA inhibits CRPC cell proliferation and induces cellular senescence.

### AA exhibits a distinct AR antagonism

Therapy resistance to antagonist treatment is in part based on AR mutations. C4-2 cells express the AR mutant T877A that mediates resistance to the AR antagonist flutamide (F). Noteworthy, F renders this AR mutant to a potent transactivator [14]. Therefore, we tested other AR mutants that are known to mediate resistance to other AR antagonists in order to obtain deeper insights into the antagonism of AA (Fig. 1i). For that purpose, we used expression vector transfection experiments in CV1 cells lacking endogenous AR and related nuclear receptors such as the glucocorticoid and progesterone receptors that may interfere with the specificity of treatment. Dihydrotestosterone (DHT), an AR agonist, is rapidly metabolised and its metabolites may act as oestrogen receptor beta agonists [15]. Hence, the much less metabolisable synthetic androgen methyltrienolone (R1881) was also used in complementary experiments to analyse AR specific mediated activity. Inhibition of wild-type AR by AA was previously demonstrated [6]. Our data suggest that AA neither activates nor inhibits the ligand-activated AR-T877A, which mediates resistance to F. Similar to the T877A mutation, the AR-W741C, which mediates resistance to bicalutamide (Bic) or AR-F876L, which mediates resistance to enzalutamide (Enz), were not activated by AA (Fig. 1i). Notably, co-treatment of DHT with AA indicates that AA counteracts androgen-induced transactivation of these mutant ARs. These data suggest that AA uses another molecular mechanism to antagonise the AR compared to these clinically used anti-androgens.

### AA inhibits growth of multicellular CRPC 3D tumour spheroid model

**Fig. 2:**
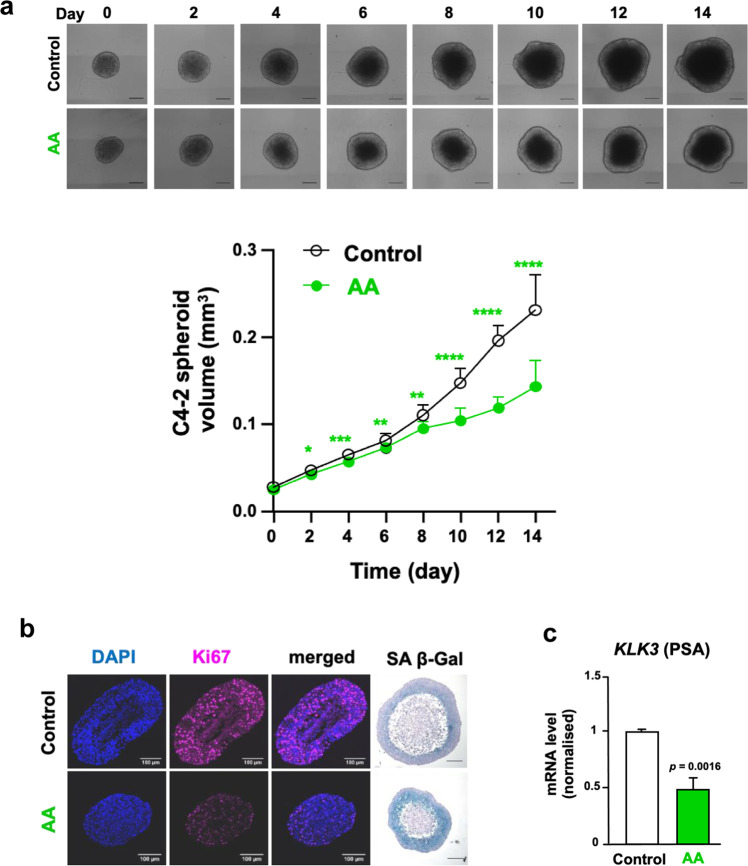
AA decreases growth of C4-2 spheroids. **a** C4-2 spheroids were generated and treated in 5% untreated FBS with 100 µM AA or DMSO as solvent control for 14 days. Medium with AA or solvent control was refreshed every 48 h. Representative spheroid pictures with scale bar 200 µm (upper panel) and quantification of spheroid growth (lower panel) are presented (*n* = 12, two-tailed unpaired student’s *t* test). Error bars indicate SEM. **b** Microtome sliced C4-2 spheroids were subject for DAPI staining of nuclei (blue), immunofluorescence for detection of the proliferation marker Ki67 (magenta), merged, and SA β-Gal activity staining. **c** qRT-PCR of *KLK3* mRNA level encoding the diagnostic marker PSA as a direct AR target gene was analysed using extracted RNA from C4-2 spheroids treated under similar condition as Fig. 3a for 8 days (control *n* = 4, AA *n* = 3, two-tailed student’s *t* test). Error bars indicate SEM.

The spheroid model is a superior tumour model in terms of complexity and drug delivery compared to monolayer cultures [16]. To analyse growth inhibition of CRPC cells by AA, a 3D tumour spheroid model from C4-2 cells were generated and treated with AA, resulting in reduced spheroid volumes (Fig. 2a). Spheroids were cryo-sliced and immunostained. DAPI-staining and Ki67 immunostaining suggest a multi-layer spheroidal structure with the proliferation layer located more at the outer layer (Fig. 2b). AA treatment reduced potently Ki67 staining and enhanced the SA β-Gal positive stained spheroidal zone (Fig. 2b), indicating the induction of cellular senescence being enhanced in a specific layer of spheroids. Similar results were obtained with LNCaP spheroids (Supplemental Fig. S1). Analysing the expression of *KLK3*, encoding PSA, confirms the repression in AA-treated spheroids (Fig. 2c). The data indicate an antiproliferative action of AA and downregulation of AR target genes in 3D spheroids of CRPC.

### Transcriptome sequencing indicates AA counteracts androgen-mediated modulation of gene expression

**Fig. 3:**
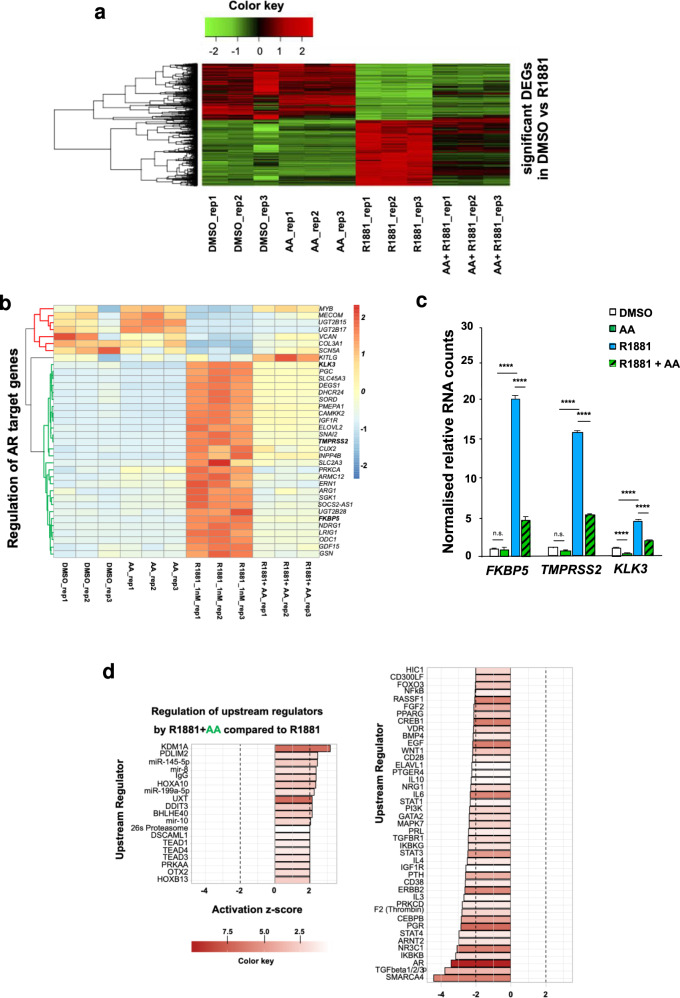
AA rescues the action of AR signalling in C4-2 cells. **a** Heat map from transcriptome analyses shows the hierarchical clustering of all identified differentially expressed genes (DEGs, adjusted *p* value < 0.05) upon AA alone, R1881 alone, AA + R1881 co-treatment, and the solvent control DMSO in all of the replicates. **b** Heat map of expression of known AR target genes plotted in all of the replicates and groups. **c** Normalised RNA counts of positively regulated AR-target genes in C4-2 cells upon AR ligand treatments. *n* = 3, for calculating the adjusted *p* value and normalised counts (DESeq2R package); *p < 0.05; **p < 0.01; ***p < 0.001; ****p < 0.0001; n.s., not significant. **d** Activity prediction of top 50 upstream regulators in R1881 + AA co-treatment compared to R1881 alone. Colour bar on the right side shows the −log 10 of enrichment *p* value (the redder, the more significant). The *x*-axis shows the activity prediction (Z-score) for each upstream regulator (using RNA-seq data, calculated by IPA). Positive *Z*-score shows activation and negative Z-score shows inhibition. Dashed line indicates the significance threshold, *Z*-score < −2 and Z-score> 2 are significant.

To verify whether AA counter-regulates androgen regulation of AR target genes, AA was co-treated with R1881 and compared to transcriptome landscape of R1881 alone using RNA-seq analyses. For each treatment three independent RNA-seq biological replicates were analysed in terms of homogeneity in expression pattern (Supplemental Fig. S2). The principal component analysis (PCA) plot shows the rescue effect of AA in the combinatory treatment (Supplemental Fig. S2). Interestingly, R1881 induces a pronounced change in the transcriptional pattern of C4-2 cells (Supplemental Fig. S2, arrow a), whereas AA in the co-treatment compared to R1881 in part rescues the R1881-induced gene expression pattern toward the control treatments (Supplemental Fig. S2, arrow b). Moreover interestingly, AA alone changes the gene expression pattern in the opposite direction of androgen. This indicates that the serum may contain a small level of androgen (Supplemental Fig. S2, arrow c). In total, R1881 upregulates the mRNA expression of 1411 (354 + 1057) genes and downregulates the mRNA expression of 1184 (706 + 478) genes. AA in combination with R1881 significantly rescues 1057 genes that are upregulated by R1881 and 478 genes that are downregulated by R1881. The heat map depicts the differentially expressed genes (DEGs) of all significantly DEGs with adjusted *p* value < 0.05 after treatment with R1881 compared to the control DMSO (Fig. 3a). Thus, androgen-mediated downregulation of genes exhibits an upregulation by AA and conversely, upregulated genes by androgens are repressed by AA (Fig. 3a). Interestingly, focusing specifically on AR-regulated genes, it suggests that almost all of the DEGs induced by R1881 were rescued by AA. Using specifically known AR transcriptionally regulated target genes, analysis by IPA software confirms in the heat map that the changes which are directly related to AR are also rescued by AA treatment (Fig. 3b) and exemplified by depicting key AR target genes such as *FKBP5, TMPRSS2 and KLK3* (Fig. 3c and Supplemental Fig. S3).

These datasets were also used to identify upstream regulators being either significantly activated or repressed (Fig. 3d). Among activated upstream regulators are epigenetic factors such as KDM1 and HOXB13 as well as the UXT chaperone. Among downregulated upstream regulators growth promoting regulators are enriched. Thus, the transcriptome analysis suggests that AA acts genome-wide as a counter-actor of androgens with emphasis on growth inhibition.

### AA inhibits CRPC xenograft tumour growth in mice

**Fig. 4:**
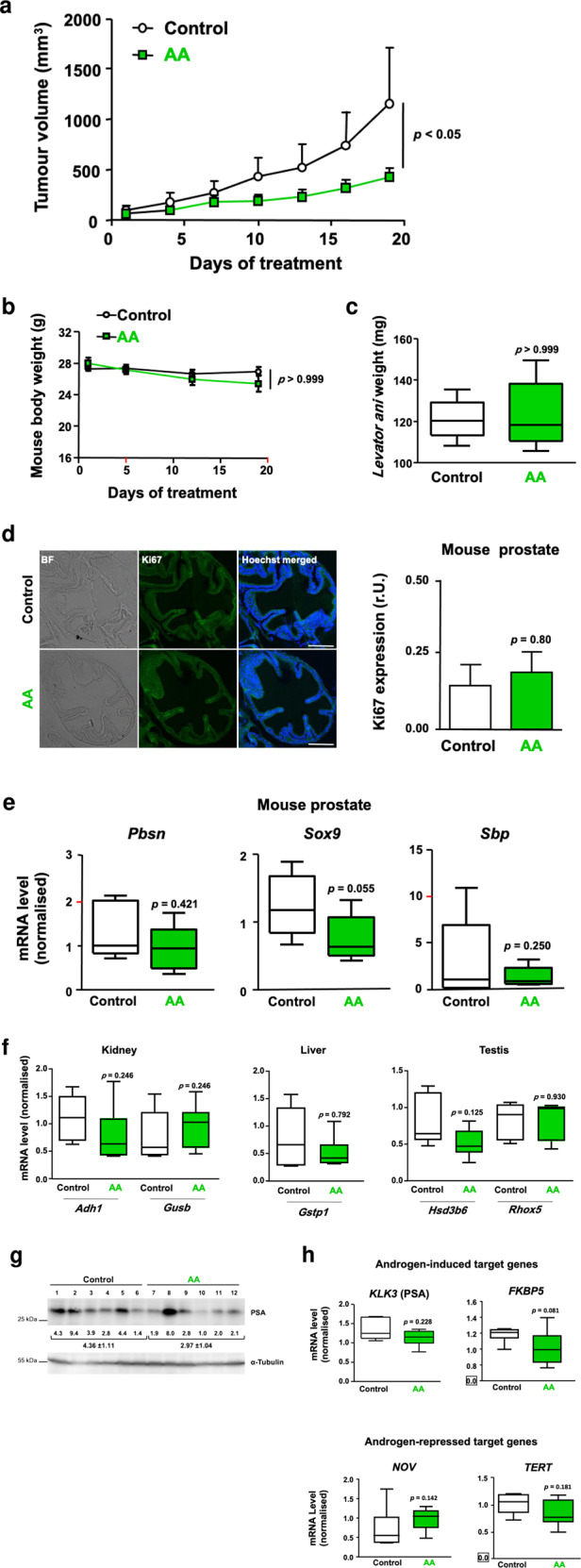
AA inhibits CRPC tumour growth in vivo. C4-2 cells were injected in each flank of immune-deficient mice. Tumour growth, tumour proliferation and mouse organs were analysed. **a** Tumour volume in AA-treated mice compared to control. Male nude mice received subcutaneous inoculation with C4-2 cells in each flank. Upon tumour formation mice were randomly grouped (control *n* = 6, AA *n* = 7) and injected daily with vehicle or AA (100 mg/kg). Tumour volume was measured every 3 days using a calliper. Two-way ANOVA test was performed. *p < 0.05, *t* = 2.790. **b** Body weight of mice was measured once a week and plotted against days of treatment. **c** The weight of *Levator ani* from control compared to AA-treated mice. **d** Representative images of immunofluorescence staining of mouse prostates. Mouse organs were isolated from mice sacrificed at day 19. Bright-field (BF) and scale bars indicate 100 μm. Relative level of Ki67 expression per cell nucleus was analysed and noted as relative units (r.U.). 20 images were analysed per tissue and treatment; *n* = 5 per treatment group, *p* = 0.80. **e** qRT-PCR analyses of various prostate-specific AR target genes for murine prostate with seminal vesicles of xenografted mice (control *n* = 5, AA *n* = 6). *Rpl13a* was used as housekeeping gene for normalisation. **f** Different organs from xenografted mice (control *n* = 5, AA *n* = 6) were analysed for organ-specific AR target gene expression by qRT-PCR. *β-Actin* was used as housekeeping gene for normalisation. Mann–Whitney *U* test was used with error bars indicating SEM. **g** Western blot analysis of PSA protein levels in the xenografted tumour tissues isolated from vehicle- or AA-treated mice (*n* = 6). The normalised protein levels are indicated along with the values underneath the brackets. **h** qRT-PCR results of extracted RNA from tumour tissues isolated from xenografts to analyse expression of AR target genes. Both *β-Actin* and *RPL13a* were used as housekeeping genes for normalisation. Mann-Whitney U test was used with error bars indicating SEM.

To analyse AA activity and CRPC tumour growth inhibition by AA in vivo, C4-2 cells embedded in Matrigel were injected as xenografts in each flank of immune-deficient male mice. Mice were either mock-treated or treated with AA (100 mg/kg/day) by intra-peritoneal injection. Tumour volume was measured during the treatment period. Compared to mock, AA treatment inhibits C4-2 tumour growth in mice (Fig. 4a). Subsequently, the subcutaneous tumours, the mouse prostates, seminal vesicles, testes, livers, kidneys and the *levator ani* were removed for analysis. The body weight of mice was not affected during the 19-day treatment (Fig. 4b). Furthermore, the weight of the androgen-responsive *levator ani* muscle tissue [17] was not altered by AA (Fig. 4c). Interestingly, the analyses of mouse prostates indicated no significant changes of Ki67 expression or prostate morphology by AA treatment (Fig. 4d). The expression of known mouse AR-target genes in mouse prostates was analysed, revealing a trend for a decrease in the medians of mRNAs encoding probasin (*Pbsn*), sex determining region Y-box 9 (*Sox9*) and spermine binding protein (*Sbp*) after AA treatment (Fig. 4e). Hence, the analysis was expanded to other mouse tissues and the expression of other known AR target genes. The expression of genes encoding alcohol dehydrogenase1 (*Adh1*) and β-glucuronidase (*Gusb*) in the kidney, glutathione S-transferase pi 1 (*Gstp1*) in the liver and hydroxy-steroid dehydrogenase 3 beta-6 (*Hsd3b6*) or reproductive homeobox 5 (*Rhox5*) in testis was not significantly affected but showed a trend towards for a decrease by AA treatment (Fig. 4f). There is also a trend of reduced PSA levels in the tumours of AA-treated mouse group (Fig. 4g). Similarly, at mRNA level the expression of *KLK3*, *FKBP5*, and *hTERT* was reduced by AA treatment (Fig. 4h). Further, the *NOV* mRNA levels, encoding a tumour suppressor, were enhanced in the tumours of AA-treated mice group. Taken together, these data support the finding that AA inhibits tumour growth in vivo and counteracts AR target gene expression in the tumours.

### AA inhibits angiogenesis

**Fig. 5:**
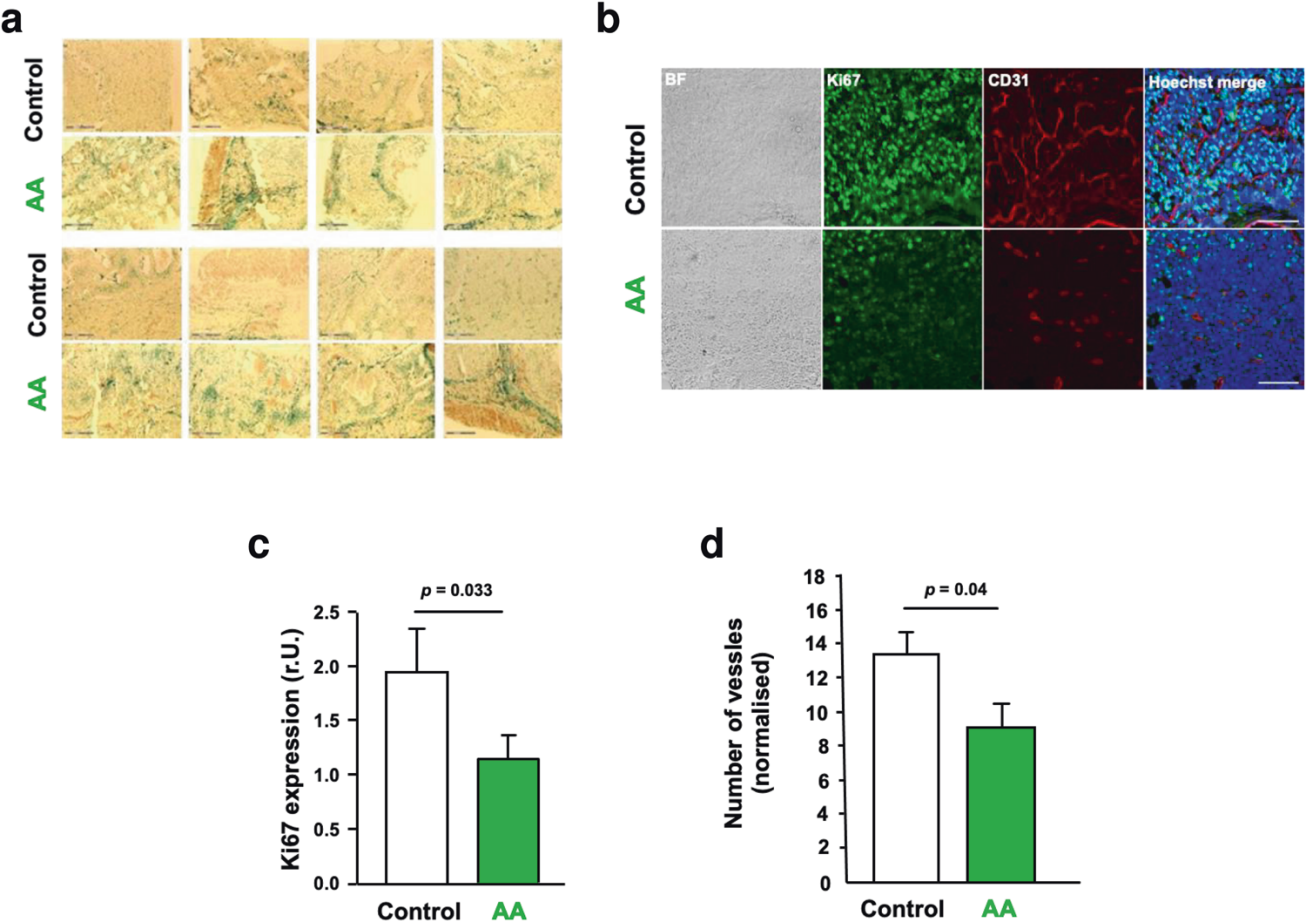
AA inhibits angiogenesis in CRPC xenografted tumours. **a** SA β-Gal staining of native tumour slices of AA and mock-treated mice. **b** Immunofluorescence staining of C4-2 tumour tissues for proliferation marker Ki67, endothelial marker CD31 and Hoechst 33258. Tumour tissues were isolated from mice that were sacrificed on day 19. Representative images are shown. BF, bright-field; scale bars indicate 100 μm. **c** Ki67 staining of tumour tissues was quantified by ImageJ software. Obtained values were plotted representing the relative level of Ki67 expression per cell nucleus analysed, noted as of relative units (r.U.). 20 images were analysed per tissue and treatment. **d** Vessel numbers were measured by quantification of CD31 staining.

The native tumour samples of the CRPC xenograft mice were further analysed for SA β-Gal activity (Fig. 5a). SA β-Gal positive cells were detected in the sliced tumour tissues indicative of senescent cells in the CRPC tumours. The proliferation marker Ki67 by immunostaining of tumour tissues slices was analysed. In line with reduced tumour size in mice and tumour growth, reduced Ki67 expression was observed in the tumour samples derived from the AA-treated mouse group (Fig. 5b, c).

Since tumour growth is dependent on neo-angiogenesis, we also analysed the levels of CD31 as a marker for endothelial cells. CD31 immunostaining of the tumours revealed a significantly reduced CD31 level in the mouse group treated with AA (Fig. 5b, d). This suggests that the number of vessels is reduced in AA-treated tumours and that AA may inhibit the neo-angiogenesis of xenograft tumours in male mice. Vessel density is considered as a very good surrogate marker of angiogenic activity and has been demonstrated as a prognostic factor in various tumours. Data suggest that vessel density is associated with higher tumour grade and stage, and worse outcome in PCa [18, 19].

**Fig. 6:**
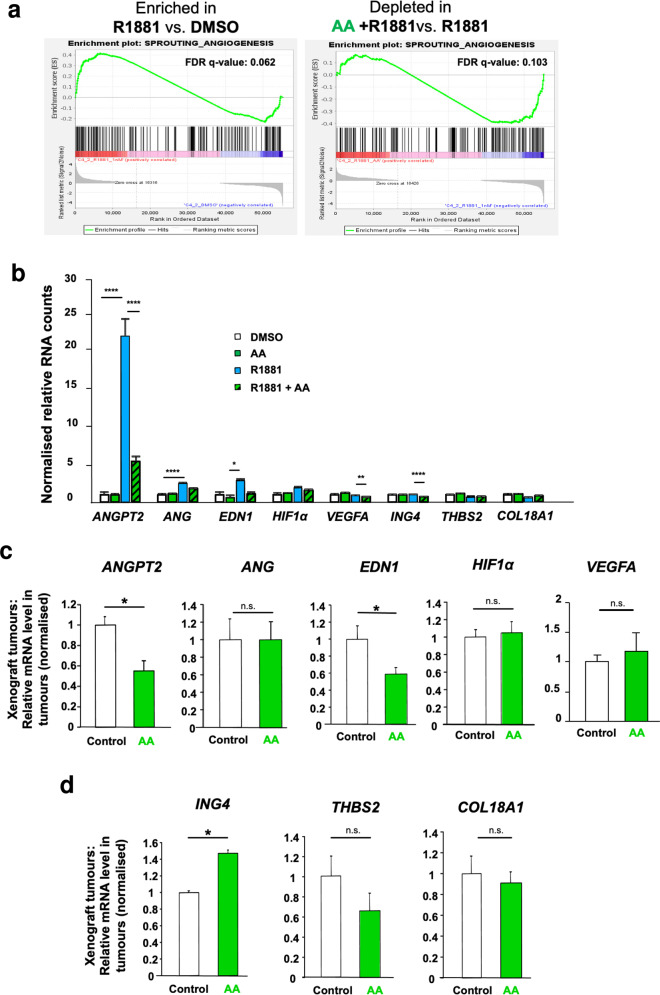
Pathway analysis of androgen and AA-modulated angiogenic factors. **a** GSEA plot of sprouting angiogenesis gene set using RNA normalised counts indicates positive enrichment of the gene set by R1881 compared to DMSO**;** FDR *q*-value= 0.062 and counteraction by AA, FDR *q* value = 0.103. **b** Regulation of mRNAs by AR ligands for specific angiogenic factors derived from RNA-seq data of androgen- and AA-treated C4-2 cells for 72 h (*n* = 3). qRT-PCR of extracted RNA from C4-2 xenograft tumours indicated expression changes of pro-angiogenic (**c**) and anti-angiogenic factors (**d**). Mice were treated under the same condition as described in Fig. 4a (*n* = 5, two-tailed student’s *t* test).

To identify if key genes of angiogenesis are over-represented by androgen treatment and depleted by AA in treated C4-2 cells, gene set enrichment analysis (GSEA) was performed. For that purpose, a custom gene set was generated by pooling seven different GO terms and eight angiogenic genes of interest. According to the GSEA recommendation, the cut-off of 25% error for FDR is considered as significant [20, 21]. The GSEA suggests a significant positive enrichment of angiogenic gene set upon R1881 treatment compared to DMSO and a significant rescue effect by AA with being negatively enriched by AA and R1881 co-treatment compared to R1881 alone (Fig. 6a). These data support that AA inhibits angiogenesis.

### AA counteracts neo-angiogenic transcriptome pathway

The hypothesis was that androgens promote and AA treatment counteracts androgen-induced angiogenic pathway. First bioinformatic analysis of the transcriptome data was employed. Notably, genes involved in sprouting angiogenesis are significantly enriched by androgen treatment (Fig. 6a). Furthermore, sprouting angiogenesis regulating genes are depleted by AA treatment (Fig. 6a) indicating that androgen induces genes that promote angiogenesis and AA counteracts androgen action. The normalised RNA counts of key genes were compared upon different treatments. Angiopoietin 2 *(ANGPT2)* was the predominant pro-angiogenic factor being strongly upregulated by androgen and significantly rescued by AA (Fig. 6b). Interestingly, other pro-angiogenic factors such as angiogenin (*ANG)*, endothelin 1 (*EDN1)* and hypoxia inducible factor α *(HIF1α)* show slight regulation by androgens (Fig. 6b). Surprisingly, we did not detect a significant androgen regulation of the vascular endothelial growth factor (*VEGFA*) at mRNA and protein levels (Fig. 6b and Supplementary Fig. S4a–c, respectively). Also, no significant change of expression inhibitor of growth 4 (*ING4)*, or Thrombospondin2 *(THBS2)* was detected (Fig. 6b). The human PCa cell line PC3, lacking AR expression, shows neither an androgen-dependent induction nor inhibition by AA of these angiogenic factors (Supplementary Fig. S4b, c) indicating AR specific regulation. Similarly, no significant regulation of ANGPT2 protein was detected in the AR-negative PC3 cells (Supplementary Fig. S4d). Thus, *ANGPT2* was detected as a prominent pro-angiogenic factor, being highly induced by androgen and significantly rescued by AA in the AR expressing C4-2 cells, indicating its AR-controlled expression.

Analysing the xenografted CRPC tumours from mice reveals that mice treated with AA downregulates the expression of the angiogenic promoting factors *ANGPT2* and *EDN1* in vivo (Fig. 6c) confirming the previous observation. The data obtained from these tumours are also consistent with an upregulation of anti-angiogenic *ING4* mRNA levels in the human xenograft tumours in vivo (Fig. 6d), with no significant changes are detected for *ANG, VEGFA, HIF1α, THBS2* and *COL18A1* (Fig. 6c, d).

To examine the effect of synthetic AR antagonists alongside with the natural compound, AA, on the expression of angiogenic factors, C4-2 cells were additionally treated with the Bic, Darolutamide (ODM-201) and Enz, which is used clinically [22]. The results show that alongside *KLK3*, *ANGPT2* is rescued by AA, ENZ, BIC and ODM-201 (Darolutamide) in co-treatment of androgen and antagonists (Supplementary Fig. S5). These results indicate that the tested AR antagonists exhibited an anti-androgenic specificity to downregulate *ANGPT2* at the transcription level.

### *ANGPT2* expression correlates with aggressiveness of PCa in patient samples

**Fig. 7:**
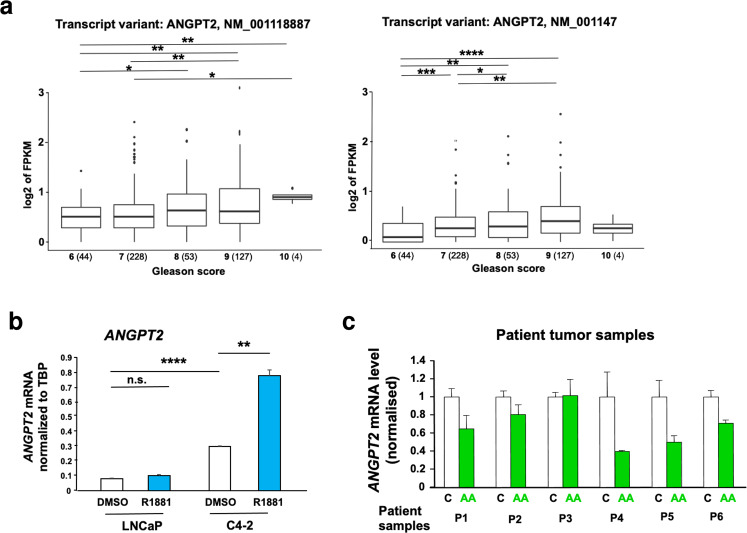
*ANGPT2* expression is associated with PCa Gleason score and is inhibited by AA in PCa patients treated ex vivo. **a** Analyses of TCGA database distinguishing the two *ANGPT2* transcript variants. X-axis represents Gleason scores of patient tissues (bold numbers). The number in parenthesis indicates the number of patient samples. Mann–Whitney–Wilcoxon test on log transformed FPKM, two tailed, *p < 0.05, **p < 0.01, ***p < 0.001, ****p < 0.0001. **b** Comparison of *ANGPT2* mRNA expression between the human castration-sensitive LNCaP and castration-resistant C4-2 cells. Cells were treated with 1 nM R1881 or DMSO as control. *TBP* served as housekeeping gene. *n* = 3; two-tailed student’s *t* test. **c** qRT-PCR analyses of *ANGPT2* levels of extracted RNA from native patient PCa samples treated ex vivo with 100 μM AA for 48 h.

To analyse the association of *ANGPT2* expression with PCa aggressiveness, two major expressed *ANGPT2* variants were investigated within different Gleason scores of PCa patient samples using the cancer genome atlas (TCGA) database. Notably, both expressed variants of *ANGPT2* (NM_001118887 and NM_001147) are highly associated with increased Gleason Scores, a measure of PCa aggressiveness. The data for theses variants suggest that increased ANGPT2 expression correlates significantly with an increased Gleason Score (Fig. 7a).

Also, *ANGPT2* mRNA expression in the CRPC C4-2 cells was compared to that of the androgen-dependent LNCaP cells, a less aggressive PCa cell line. The results indicated that *ANGPT2* levels are much higher in C4-2 compared to that of LNCaP cells (Fig. 7b). Interestingly, *ANGPT2* was inducible by androgen only in C4-2 cells (Fig. 7b).

To determine whether AA can downregulate *ANGPT2* expression in patient samples, native human PCa tumours were obtained from patients with informed consent by radical prostatectomy and treated ex vivo with AA for 2 days. The obtained data suggest that AA downregulates *ANGPT2* expression in a majority of human tumour samples ex vivo (Fig. 7c).

### ANGPT2 protein is AR ligand-dependently secreted

**Fig. 8:**
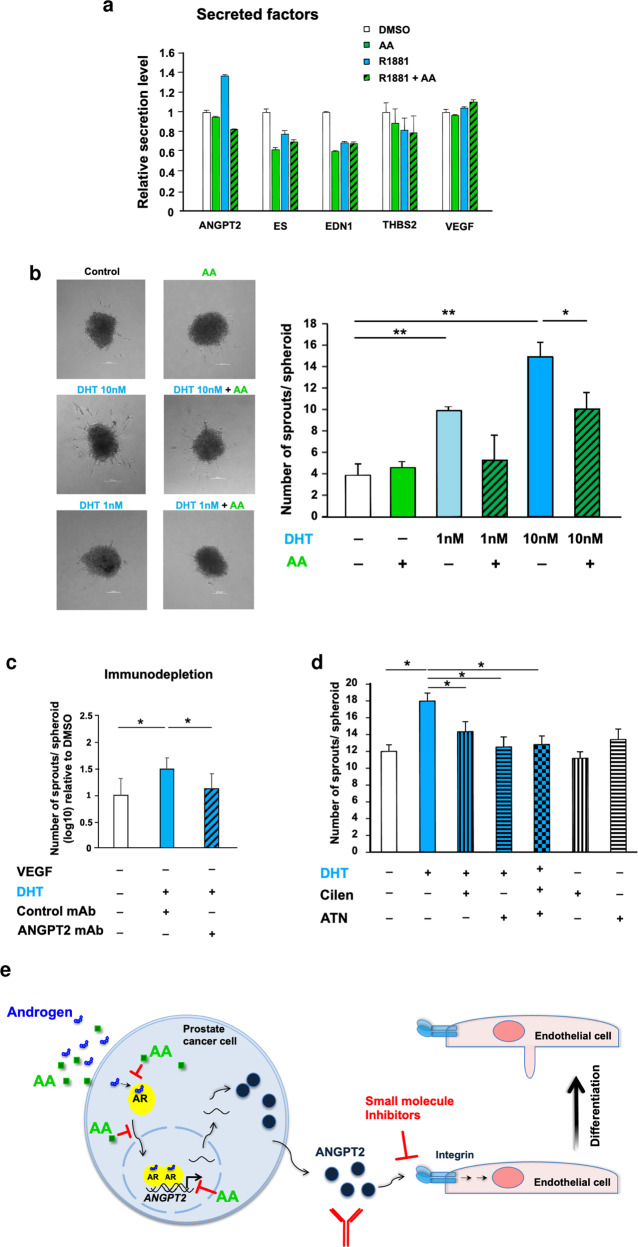
AA inhibits in vitro angiogenesis. **a** Human angiogenesis antibody arrays of serum-free CM of C4-2 cells identify the ligand-regulated secretion levels of angiogenic factors. Cells treated with 1 nM R1881, 100 µM AA, combination of R1881 and AA, or DMSO as solvent control for 72 h and followed by 2 days in serum-free medium without AR ligands. *n* = 4, two-tailed student’s *t* test. **b** Sprouting assays and invasion analysis of non-immortalised primary HUVEC spheroids embedded in collagen matrix and treated with C4-2 CM. C4-2 cells were treated as described above in (**a**). In addition, 1 nM DHT is used. *n* = 3, two-tailed student’s *t* test. Left panel: representative spheroid pictures, right panel: quantification of sprouts per spheroid. **c** Non-immortalised primary HUVEC spheroids were treated with CM of C4-2 cells that were immune-depleted from ANGPT2 using antibody coupled beads. *n* = 3, two-tailed student’s *t* test. **d** Non-immortalised primary HUVECs spheroids co-treated with C4-2 CM in combination with the indicated integrin inhibitors verified by two additional independent experiments. Two-tailed student’s *t* test; *p < 0.05. **e** Summary picture with the proposed molecular mechanism. Androgen-induced neo-angiogenesis by CRPC can be inhibited at three levels: Through (I) blocking AR by AA treatment, that (Ia) competes for androgen binding, (Ib) inhibits nuclear translocation and (Ic) chromatin binding of AR, thus reducing the expression and secretion of ANGPT2, (II) immune-blocking of ANGPT2 and (III) inhibition of ANGPT2 receptors (integrins) by small molecule inhibitors on primary human endothelial cells.

To analyse whether the secretion of angiogenic factors by C4-2 cells is promoted by androgen treatment, we performed angiogenesis protein arrays and Mass spectrometry analyses. To this aim, C4-2 cells were treated in serum-free medium with AR ligands and after several washing steps, the secreted factors in the C4-2 conditioned media were detected. The results indicate that the ANGPT2 protein is an androgen-induced secreted factor (Fig. 8a and Supplementary Fig. 6a, b). The androgen-induced secretion of ANGPT2 was counteracted by AA when co-treated with androgen (Fig. 8a and Supplementary Fig. S6a). Interestingly, secretion of VEGF, classically known as a key factor for angiogenesis [23], was neither induced by androgen nor reduced by AA (Fig. 8a). Similarly, THBS2 as an anti-angiogenic factor had not shown any regulation pattern upon the AR ligands treatments. Other angiogenic factors such as EDN1 and endostatin (ES) were reduced by all the treatments compared to control (Fig. 8a). In line with this, analyses of cytokine arrays also reveal inhibition of ANGPT2 secretion by C4-2 cells upon AA treatment (Supplementary Fig. S6c, d). Notably, the secretion of ANGPT2 is significantly inhibited not only by AA but also by the second-generation AR antagonist Enz (Supplementary Fig. S6d). Taken together, ANGPT2 is the main secreted angiogenic factor that the secretion being induced by androgen and inhibited by AA treatment.

All together, these data suggest that androgen regulates angiogenic factors not only at transcriptional level but also at secretion level. AA treatment counteracts androgens for ANGPT2 upregulation and secretion. Thus, it is suggested that ANGPT2 may be one key factor that promotes tumour neo-angiogenesis and being downregulated by AA in vivo and in vitro.

### ANGPT2 as a key angiogenic factor in the induction of sprouting

To confirm a functional relevant upregulation of angiogenesis by androgen treatment and counteraction by AA, we used two established in vitro angiogenesis model systems, the tube or branch formation and sprouting assay of primary human endothelial cells. The hypothesis was that ANGPT2 is secreted from C4-2 cells and induces sprouting as a key factor of neo-angiogenesis.

For this aim, the C4-2 secretome was analysed in protein arrays and ANGPT2 was identified as a secreted factor. The secretion is enhanced by androgen and counteracted by AA (Fig. 8a). Next, non-immortalised primary human umbilical vein endothelial cells (HUVECs) were incubated with serum-free C4-2 conditioned media (CM). The tube formation assay revealed an enhanced branch number and branch length of HUVECs due to androgen treatment. Treatment with AA inhibits the androgen-induced branch length, suggesting that AA counteracts functionally androgen-induced endothelial cell branching (Supplementary Fig. S7). To analyse sprout formation of endothelial cells by CM in a 3D environment, we generated spheroids from non-immortalised primary HUVECs. The advantage of this system is that cells are in direct contact, which promotes cell-cell signalling among endothelial cells. For that purpose, HUVEC spheroids were treated with serum-free CM from pre-treated C4-2 cells. Sprouting of HUVEC spheroids, depicted as sprouts per spheroid, is enhanced by the CM pre-treated with androgen (Fig. 8b). As expected, the enhanced androgen-induced sprouting is downregulated when co-treated with AA (Fig. 8b). These observations further confirm that secreted factors from CRPC C4-2 cells upregulate sprouting angiogenesis of primary human endothelial cells and indicate that androgens promote, whereas AA counteracts, neo-angiogenesis of non-immortalised human primary endothelial cells.

Next, we addressed the question of whether ANGPT2 protein, secreted in an androgen-dependent manner by C4-2 cells, is the key factor functionally mediating the sprouting of HUVEC spheroids. For that purpose, the serum-free CM of C4-2 cells were immunodepleted using an ANGPT2 antibody coupled to beads. Sprouting assays using primary HUVEC spheroids treated with ANGPT2-depleted CM indicate that the androgen-induced sprouting is inhibited by ANGPT2 immunodepletion of the CM (Fig. 8c). These data strongly suggest that ANGPT2 seems to be a major pro-angiogenic factor secreted by C4-2 cells.

To confirm this functional observation, two inhibitors of the known ANGPT2 receptors, integrins α_v_β_3_, α_v_β_5_ and α_5_β_1_ [24], were used in sprouting experiments. Cilengitide, an inhibitor of integrin α_v_β_3_ and α_v_β_5_ that may be used as an agent to reverse drug resistance induced by α_v_β_3_ [25]. The anti-angiogenic ATN-161 compound blocks the integrins α_5_β_1_ and α_v_β_3,_ leading to reduced neo-vascularisation [26]. These compounds were employed to treat HUVEC spheroids with CM of C4-2 cells. The results suggest that both inhibitors alone and in combination significantly rescue the DHT-induced sprouting (Fig. 8d). Thus, the depletion of ANGPT2 itself, the inhibition of ANGPT2 receptors by small inhibitor compounds, or treatment with AA block androgen-induced sprouting, highlighting the notion that ANGPT2 is the key angiogenesis-promoting factor of the C4-2 cell secretome.

Taken together, these data suggest that androgens promote neo-angiogenesis in CRPC tumours through secretion of ANGPT2 and that AA counter-regulates this process. These findings suggest that AA serves as an inhibitor of androgen-induced neo-angiogenesis of human PCa and provide evidence for ANGPT2 and its receptors as potential key drug targets (Fig. 8e).

## Discussion

CRPC is known to be dependent on AR signalling, despite the cells being resistant towards removal of androgens such as by ADT. CRPC cells and tumours will eventually become therapy-resistant even to a full-blockade of AR through ADT and second-generation AR antagonists co-treatment via adaptive signalling [27], which may include a variety of different pathways that activate AR signalling or may select for those AR mutants that not only overcome antagonism but are instead rather strongly activated by a specific AR antagonist. These and other adaptive responses are presumably the major problems in the treatment of therapy-resistant CRPC. Nevertheless, any growing tumour requires neo-angiogenesis, which provides a potential for a combinatorial therapy targeting pro-angiogenic factors in concert with inhibition of AR signalling.

The data suggest that AA exhibits a distinct mechanism for AR antagonism compared to F, Bic and Enz. Chemically, all three latter compounds have a methyl-trifluoride substitution. In contrast to F, which possesses only one benzene ring, Bic and Enz have two benzene rings with one halogenated aromatic ring. AA consists of only one aromatic benzene ring but has no halogenated substitutes. Interestingly, this small non-steroidal chemical compound inhibits the wild-type AR itself and additionally those AR mutants that mediate resistance to the AR antagonists F, Bic or Enz. Although all these AR ligands bind to the AR ligand-binding pocket and inhibit the N/C interaction of AR, the detailed underlying structural changes of AR to mediate AR antagonism is unknown. Like these AR antagonists, AA inhibits the binding of androgens to AR, however, AA exhibits a specific mode of AR antagonism being distinct from the above-mentioned clinically used antagonists. However, AR antagonists do not just inactivate AR by turning it to a non-functional receptor, rather anatagonists trigger cellular senescence in CRPC.

RNA-sequencing transcriptome data, as well as the analysis of the secretome by protein arrays, indicates that the secretion of VEGF is not regulated by AR. Therefore, the used cell line provides a suitable model system to analyse non-VEGF angiogenic pathways in PCa. Our data suggest that ANGPT2 appears to play a crucial role in angiogenesis. This is in line with data showing that blocking ANGPT2 with neutralising antibodies leads to a reduction in mouse retinal vessel area.

It is known that high concentrations of ANGPT2 enhance the survival of endothelial cells through phosphatidylinositol 3′-kinase/AKT [28], which is an essential mechanism during angiogenesis. Our data suggest that the pro-angiogenic factor ANGPT2 is an androgen-upregulated and androgen-promoted factor secreted from CRPC cells. Increased ANGPT2 expression correlates with increased Gleason Score as well as with more invasive potential in other cancer types, such as disease progression of metastatic melanoma [29], metastasis to the lymph node of breast cancer [30, 31], and with worse prognosis and poor survival in lung cancer patients [32, 33].

It is generally assumed that the main function of ANGPT2 is the partial antagonism of ANGPT1, inhibiting its binding to Tie-2 receptor [34, 35]. However, in endothelial cells a pro-angiogenic role of ANGPT2 through an integrin axis and a Tie-2-independent mechanism was described [24, 36, 37] as promoting cancer invasiveness [30], therefore, being considered as an important therapeutic target. Further studies show that the ANGPT2 and integrin receptors (α_v_β_5_ and α_v_β_3_) promote angiogenic pathways [36]. Integrin α_v_β_3_ was characterised as a marker of angiogenic vascular tissue and its enhanced endothelial expression was observed on blood vessels in human tumour biopsies but not on normal vessels in human tissues [37–39]. Our data provide the basis that the inhibition of these cognate integrins by small molecule inhibitors in primary HUVECs represses sprouting even in the presence of ANGPT2 in CM from CRPC cells.

Of the several integrin inhibitors, cilengitide and ATN-161 are well tolerated and less toxic in clinical trials [40]. Cilengitide is characterised as one of the first anti-angiogenic small molecules to enhance radiotherapy in clinical trials [41]. ATN-161 in combination with chemotherapy reduces metastasis and improves survival in mice with colon cancer [42]. Since integrins compensate for each other, in our study we have used a combination of two anti-angiogenic compounds cilengitide, which inhibits α_v_β_3_ and α_v_β_5_ [43] and ATN-161 which blocks integrin α_5_β_1_ and binds integrin α_v_β_3_ [44]. The combination treatment with both small molecule compounds revealed a significant reduction in endothelial cell sprouting but no synergistic effect indicating a maximal blockage of ANGPT2-mediated sprouting, perhaps through the overlapping target of both compounds, the integrin α_v_β_3_.

Our data provide a basis for an ANGPT2-integrin pathway in endothelial differentiation as well as for sprouting through the androgen controlled secretome of PCa. The androgen-regulated expression at a transcriptional level and the androgen-induced secretion of ANGPT2 is inhibited by AA. In line with this, AA reduced the number of vessels in CRPC xenograft tumours in immune-deficient male mice. It should be noted that ANGPT2 not only drives tumour neo-angiogenesis but also plays a crucial role in the induction of inflammation in the tumour microenvironment. ANGPT2 prompts the infiltration of myeloid cells in an integrin-dependent manner [45]. It also enhances inflammation by sensitising endothelial cells to TNF-α [46]. However, using immune-deficient mice, the underlying mechanism of reduced tumour volume and vessel numbers due to AA treatment, might be rather the changed secretome by the tumour cells, including inhibition of ANGPT2 secretion by AA. This is supported by the observation that the inhibition of secreted ANGPT2 signalling either by an antibody used for immune-depletion or blocking the integrin receptors for ANGPT2 through small molecule inhibitors.

Clinical trials that focused on the inhibition of VEGF signalling have not been adequately successful so far and lack a benefit in improving overall survival [47]. One possibility is that the potency of VEGF signalling for angiogenesis may change during PCa tumour evolution and VEGF might therefore not always be the major factor and drug target for neo-angiogenesis. In PCa, several studies demonstrated an association of elevated levels of VEGF and higher Gleason scores, PSA levels, clinical progression and inferior survival [9, 48–51]. Of note, CRPC is a heterogenous disease with vastly differing mechanisms to achieve androgen independent cell growth with a presumably heterogenous composition of castration-resistant cell subtypes. This may also account for the different observations of VEGF regulation by androgens and implies that a subset of cells within the CRPC tumour regulate angiogenesis by different mechanisms. We provide here evidence and a model system of a VEGF-independent pathway of androgen-regulated angiogenesis.

C4-2 cells used as a model system for CRPC express high levels of AR, a characteristic of castration resistance (Raclaw et al., 2008). Comparing the CRPC cell lines C4-2 with PC3, the VEGFA protein level in both cell lines is not changed by androgen treatment (Supplementary Fig. S4b), which is expected for the AR negative PC3 cells.

Bevacizumab is a humanised immunoglobulin G1 monoclonal antibody that binds and inhibits the biologically active isoforms of human VEGF. This agent has shown to be effective in several malignancies such as colorectal, breast, non-small cell lung and kidney cancer [52]. However, the clinical trials unfortunately did not show a clear improvement in overall survival.

Interestingly, *ANGPT2* is transcriptionally upregulated after VEGFR2 blockade. Thus, therapy-induced upregulation of ANGPT2 potentially drives tumour resistance to VEGFR2 blockade [53]. Using a model system with strongly reduced VEGF signalling, we identified ANGPT2 as a major pro-angiogenic factor, which reveals a potential novel drug target.

In the normal adult, ANGPT2 is predominantly present in the placenta and uterus, at sites of vascular remodelling [54]. ANGPT2 can be secreted rapidly after a stimulus and has a long half-life of more than 18 h [55]. Recently, ANGPT2 was identified as a major pro-angiogenic factor in a subset of melanomas [56]. In many other cancer types, ANGPT2 has been suggested as a pro-cancerous factor [57]. However, for PCa the body of evidence for ANGPT-induced angiogenesis is weak. Although in a small cohort, *ANGPT2* mRNA levels were not detected to be differentially expressed in hormone-refractory PCa compared to pT2 and pT3 PCa [58], ANGPT2 expression seems to be associated with a high PCa Gleason score and thus with cancer aggressiveness.

The data suggest that ANGPT2 is one of the key angiogenic factors of C4-2 cells, however this does not rule out other important angiogenic factors in PCa neo-vascularisation [59]. It is possible that PCa may activate distinct angiogenic pathways, depending on tumour evolution and treatment, which makes it difficult to rely only on one angiogenic inhibitor.

We show that ANGPT2-mediated sprouting angiogenesis occurs through ANGPT2 via binding to the integrins α_v_β_3_, α_v_β_5_ and α_5_β_1_ of endothelial cells, also known as ANGPT2 receptors [24]. Inhibition of these integrins with small molecules such as cilengitide and ATN-161, in order to block tumour angiogenesis, has been evaluated as promising in recent clinical trials [60]. To our knowledge, no previous research has investigated the regulation of ANGPT2-mediated angiogenesis by androgen in CRPC. Thus, we provide evidence that the pro-angiogenic activity of ANGPT2 in CRPC can be blocked at three levels, by antagonising AR, through anti-ANGPT2 antibodies and through small molecule inhibitors for ANGPT2 receptors (Fig. 8e).

## Material and methods

### Cell culture, growth and reporter assays

Male primary human umbilical vein endothelial cells (HUVECs, ethic committee approval number 2018-1052-BO) were isolated from new-born babies, which provide more comparable results to in vivo vasculature compared to commercially available endothelial cells that have been moderately modified and pre-selected to improve proliferative capacity. HUVECs were cultured in endothelial cell growth medium (ECGM) from PromoCell (C-22010). General procedures for culturing the castration-resistant prostate cancer (CRPC) C4-2 and PC3 as well as the CSPC LNCaP cells including growth assays were performed as described earlier [61]. The AR mutants and luciferase reporter assays were performed as previously described [62].

### Generation of C4-2 and LNCaP spheroids

Spheroids were generated by forced floating method [63] in 96-well ULA plates (1000 C4-2 cells/well) 24 h after seeding, AR ligands or DMSO diluted in fresh medium was added to the spheroids. For each treatment, six technical replicates were conducted. The treated spheroids were incubated for the indicated number of days with the medium being replaced every 72 h. For that purpose, half of the old medium was discarded and fresh medium containing AR ligands or DMSO was added.

### Mouse studies

Experiments were approved by the Thüringer Landesamt für Lebensmittelsicherheit und Verbraucherschutz, Germany (02-006/11 and 2019-1502). C4-2 cell suspension (10^6^ cells per 50 μl 1× PBS) was mixed 1:1 with BD MatrigelTM (BD Biosciences, Heidelberg, Germany) and injected subcutaneously (s.c.) into both flanks of the intact (non-castrated) nude mice (5-week-old male athymic nude mice, Harlan Laboratories, Rossdorf, Germany). After the tumours reached a size of approximately 80 mm^3^, mice were treated with vehicle or AA (100 mg/kg) daily by intra-peritoneal (i.p.) injection. Tumour size was measured every 72 h using a caliper (tumour volume = (length × width^2^) × 0.56). Mice were weighted once a week and sacrificed after 19 days or if weight loss exceeded 20% of initial weight. For immunofluorescence analysis of CD31, 7–14 pictures per mouse were analysed (control *n* = 4 and AA *n* = 3). Graphpad was applied to conduct two-tailed student’s *t* test. Outliers were recognised and removed.

### Senescence-associated beta-galactosidase (SA β-Gal) assays

Senescence assays were performed as described previously [63].

### Reverse transcription quantitative real-time PCR (qRT-PCR)

qRT-PCR assays were performed as explained elsewhere [64]. The primer sequences are listed in Supplementary Tables 1 and 2.

### Antibodies and Western blot analyses

Preparation of whole cell lysates and Western blotting were performed as described elsewhere [65]. For protein extraction from mice organs, sections (≤50 mg) were crushed with a pestle in liquid nitrogen and suspended in 400 μl of ice-cold lysis buffer (50 mM Tris pH 7.4, 150 mM NaCl, 1 mM EDTA, 1% Triton-X100, 1 mM Na_3_VO_4_, 1 mM NaF, 2.5 mM Na-phosphate buffer (77.4 mM Na_2_HPO_4_, 22.6 mM NaH_2_PO_4_, pH 7.4, 1 mM PMSF) containing 1× complete protease inhibitor tablet (Roche, Germany). After ultrasonification in a 4 °C water bath for 30 min, samples were incubated on ice for 10 min and then centrifuged (21,500 × *g*, 2 min, 4 °C). The primary antibodies used for immunodetection were PSA (Cell Signaling, 2475), pRb (Abcam, ab6075), phospho (S780)-pRb (Cell Signaling, 9307), phospho (S807/811)-pRb (Cell Signaling, 9308), VEGFA (ABclonal, A5708), α-Tubulin (Abcam, ab15246) and β-Actin (Abcam, ab6276). As secondary antibodies, horseradish peroxidase-conjugated anti-mouse IgG (Santa Cruz, sc-2005) or anti-rabbit IgG (Santa Cruz, sc-2370) were used. The quantification of proteins of interests relative to their α-Tubulin or β-Actin loading controls was performed using LabImage 1D software (Kapelan Bio Imaging solutions, Leipzig, Germany).

### Immunofluorescence staining

PCa tumour tissue samples were stored in 1× PBS + 0.02 % NaN_3._ For dehydration, they were incubated in 30 % saccharose in 1× PBS + 0.02 % NaN_3_ overnight. Samples were submerged in Tissue-Tek® O.C.T. compound (Sakura, Germany) and slowly frozen on dry ice. Twenty micrometres tissue sections were cut and placed on glass slides for immunofluorescence analysis. The slides were washed with 1× PBS and blocked using blocking solution (5% untreated goat serum (NGS), 1× PBS-T (1× PBS, 0.25 % Triton-X100)) for 1 h and rinsed once with 1× PBS for 5 min. For staining spheroids, permeabilisation was performed with 1x PBS containing 0.2% Triton-X100 (Roth, 3051.1) for 10 min. Primary antibodies were applied to the sections overnight at 4 °C. The primary antibodies applied were Ki67 (Vector Laboratories, VP-RM04) and CD31 (BioLegend, MEC13.3). Simultaneously, slides were incubated without primary antibodies to determine the background fluorescence of the secondary antibodies for quantification purpose. Slides were washed three times with 1× PBS at 5 min. Secondary antibodies were applied to the sections for 90 min in the dark at room temperature (RT). Secondary antibodies were anti-rabbit Alexa Fluor 488 (Invitrogen, A-11008) and anti-rat Alexa Fluor 555 (Invitrogen, A-21434). Slides were washed once with 1× PBS for 5 min and nuclei were stained with Hoechst 33258 (Molecular Probes, Darmstadt, Germany; dilution: 1:10,000 in 1× PBS) for 15 min at RT. Fluorescence signals of Ki67 were quantified using ImageJ software and the background signal from the secondary antibody was subtracted. Relative quantification represents the level of expression of Ki67 protein per nuclei analysed. Image analyses of the vessels were performed using the AxioVision image analysis software (Rel. 4.8.2; Zeiss). For that purpose, depending on the size of each tissue section, up to 14 randomly selected measurement fields from each section were photographed. All CD31-stained vessel structures in the images were counted by the examiner. Vessel density was defined as the mean number of vessels per area. In order to stain the spheroid cross-sections, an additional blocking step with 5% untreated goat serum (Biozol) for 1 h at RT was performed prior to the incubation with primary anti-Ki67 antibody (Vector VP-RM04, 1:200 dilution) overnight at 4 °C. The spheroids were washed three times with 1× PBS for 10 min. The secondary antibody anti-rabbit IgG Cyanine5 (Thermo Fisher A10523, 1:1000 dilution) were added and incubated for 1 h at RT in the dark. Samples were washed and nuclei were stained with DAPI solution (1 μg/ml in 1× PBS) for 10 min. The microscope slides were washed once with 1× PBS for 10 min and rinsed with Milli-Q water. Subsequently, spheroid slices were covered with Fluoromount-G (Southern Biotech) and cover slips. The stained spheroid slices were analysed with a confocal laser scanning microscope (Carl Zeiss, Objective: 20×/0.8 M27).

For detection of senescence-associated heterochromatin foci (SAHF), DAPI staining and detection (at 457 nm) of spheroids were performed as stated elsewhere [63]. Pictures were taken by the confocal scanning fluorescence microscope (Zeiss LSM 880 Germany).

### RNA-sequencing and transcriptome analysis

Total RNA was isolated from C4-2 cells treated for 72 h with solvent control (DMSO), 100 µM AA, 1 nM androgen R1881 and co-treatment of androgen and AA in triplicate, using peqGOLD TriFast (Peqlab, Erlangen, Germany) according to the manufacturer’s protocol. RNA samples were further processed for Poly-A RNA-seq library preparation according to the manufacturer’s instructions (TruSeq^R^RNA Sample preparation v2, Illumina). Library concentrations were evaluated on Qubit 3.0 (Thermo Fisher Scientific) and quality by using the Fragment Analyzer using the High Sensitivity NGS Fragment Analysis Kit (1 bp–6,000 bp) (Advanced Analytical). The prepared libraries were sequenced using NextSeq® 500/550 High Output Kit v2.5 (75 cycles) on the machine NextSeq500 (Illumina). The RNA counts were normalised based on the geometric library size factors. The DESeq2R package (1.20.0) with the default parameters was used.

Fastq files quality check was performed using FastQC (v0.11.5). The first 4 nucleotides were removed from the sequenced reads using fastx_trimmer (FASTX Toolkit 0.0.13). The low-quality nucleotides from the end of each read were removed using fastq_quality_trimmer (FASTX Toolkit 0.0.13) with -Q 33 -t 20 -l 25 parameters. Only the first sequenced read form the pair end (R1) was used for the downstream analysis. The fastq files were mapped to the hg19 genome using tophat (v2.1.0) with the following parameters -bowtie1 -no-coverage-search -a 5. The number of reads covering each gene was calculated using htseq-count (0.11.2) with -s no -a 0 -t exon -m intersection-nonempty parameters and hg19 gencode.v19 annotation. For further analyses all rRNA genes (5srRNA, rRNA, mt-rRNA) were removed from the count data. For calculating the *p* value and normalised count (based on the geometric library size factors), the Deseq2R package (1.20.0) was used with the default parameters (using paired test). For plotting the expression, a normalised count was used. For functional and pathway analysis, Deseq2 differential expression results (adjusted *p* value < 0.05) were analysed with IPA (Ingenuity Pathway Analysis v45868156). GSEA (Gene set enrichment analysis) was performed using GSEA (v4.0.3) software [20] with the following parameters: number of permutations = 1000, permutation type = gene_set, Enrichment statistics = weighted and Metric for ranking genes = Signal2Noise. A custom gene set of angiogenesis was generated by pooling seven different gene ontology (GO) gene sets downloaded from the https://www.gsea-msigdb.org/gsea/msigdb/index.jsp. The pRb pathway was analysed using WikiPath Retinoblastoma gene in cancer Homo sapiens. For the GSEA, a custom angiogenesis gene set was generated by pooling gene sets GO:1903672, 0002042, 0090050, 1903670, 0001569, 0090049, 1903589.

For z-score based GSEA, normalised counts (for each gene in all of the samples) were scaled using scale function in R (with centre = TRUE, scale = TRUE parameters). The average of z-scores were calculated for each group and used for drawing plot and *p* value calculation. The *p* value was calculated using two-tailed Wilcoxon paired test comparing each gene in different groups. All the gene sets were downloaded from molecular signature database (MSigDB, v7.2). The gene sets are:

ACOSTA PROLIFERATION INDEPENDENT_MYC_TARGETS_UP (PMID:18838534),

BENPORATH_PROLIFERATION (PMID; 18443585),

ZHONG_PFC_C8_ORG_PROLIFERATING (PMID: 29539641),

TOMLINS_PROSTATE_CANCER_DN (PMID: 17173048),

BILD_MYC_ONCOGENIC_SIGNATURE (16273092), WP_RETINOBLASTOMA_GENE_IN_CANCER,

TANG_SENESCENCE_TP53_TARGETS_DN (PMID:17533371),

SENESE_HDAC1_TARGETS_UP (PMID: 16250917),

SENESE_HDAC2_TARGETS_UP (PMID: 16250917),

SENESE_HDAC3_TARGETS_UP (PMID: 16250917).

### Human angiogenesis arrays

C4-2 cells (10^5^ cells/ml) were seeded overnight in DMEM containing untreated 5% FBS in six-well plates. The following day, wells were washed twice with 1× PBS and treated with DHT 10 nM or DMSO in charcoal-treated 5% FBS for 72 h. At the second day, AR ligands were refreshed. After 72 h of AR ligand treatment, the cells were washed twice with 1× PBS and incubated in serum-free DMEM for an additional 48 h. Thereafter, serum-free CM was harvested and centrifuged at 3500 rpm, 37 °C for 15 min. The array experiments were performed using the Proteome Profiler^TM^ Human Angiogenesis Antibody Array according to the manufacturer’s protocol (R&D system, ARY007). The spots were quantified using protein array analyser for ImageJ and analysed according to manufacturer’s protocol.

### Human cytokine arrays

The human cytokine antibody array C1000 kit (RayBiotech, AAH-CYT-1000) was used and the array experiments were performed according to the manufacturer’s protocol. C4-2 cells were seeded at a density of 5 × 10^5^ cells per 10 cm culture dish. After 48 h, cells were treated with 100 μM AA, 1 μM Enzalutamide (Enz) or DMSO-control for 72 h in 5% untreated FBS in DMEM. The cells were washed twice with 1x PBS and treated with serum-free DMEM and incubated for 48 h. The serum-free CM were collected and filtered with 0.2 μm filter (Sarstedt, 83.1826.001) to avoid interference of residual cells. Each membrane was incubated for 5 h at RT with 1.5 ml serum-free CM. Detection of the signals was performed using ImageQuant^TM^ LAS 4000 (GE Healthcare) and quantification of the signals was performed with LabImage 1D software (Kapelan Bio-Imaging). The quantified signals were normalised to positive controls and further analysed by IPA.

### Ex vivo treatment of prostate cancer samples

Samples were taken from patients with radical prostatectomies and treated as described previously [6] (with ethical approval (3286-11/11 and (2019-1502)) by Jena University Hospital, Germany).

### Tube formation assays

To harvest CM, C4-2 cells were first incubated for 72 h with AR ligands, the media were removed, cells were washed and ECGM was added for further 48 h. Cell supernatants (conditioned medium, CM) were collected by centrifugation to remove cell debris and these CM were used to treat HUVECs monolayer on collagen. To this aim, 10 µl collagen 3 mg/ml (Roche, 11179179001) was added to each well of µ-Plate Angiogenesis 96-Well plate (ibidi, 89646) and incubated for 3 h at 37 °C. After the collagen was solidified, 15000 HUVECs (passage 3) were dissolved in 60 µl of C4-2 CM (ECGM-based) and seeded on collagen per each well of the 96-well plate. The plate was incubated at 37 °C and 5% CO_2_ for 4 h. Pictures of tubes were taken by JuLITMStage microscope at 4 h after seeding and further analysed by ImageJ angiogenesis analyser tool.

### Sprouting assays

Sprouting assays were performed according to the protocol of Korff [66]. Two thousand cells (HUVECs, passage 3) were seeded in methocell (ECGM containing 20% methyl cellulose (Merk, M7027)) into each well of the round bottom 96-well ultra-low attachment (ULA) plate (Perkin Elmer, 6055330). Then, the plate was centrifuged at 500 rpm at RT for 10 min and incubated overnight (18 h) at 37 °C and 5% CO_2_. The next day, spheroids were harvested and embedded in a mixture of rat tail collagen type I and 20% methocell (1:1) containing a final concentration of 2% untreated FBS and 1.5 mg/ml collagen. In parallel, C4-2 cells (10^5^ cells/ml) were seeded overnight (24 h) in DMEM containing untreated 5% FBS in 6-well plate. The following day, wells were washed twice with 1× PBS and treated with 1 nM and 10 nM DHT, 100 µM AA or DMSO in charcoal-treated 5% FBS for 72 h. At the second day, AR ligands were refreshed. After 72 h treatment, the cells were washed twice and incubated in serum-free DMEM for additional 48 h. Thereafter, the serum-free CM were harvested and centrifuged at 3500 rpm, 37 °C for 15 min. The collagen embedded spheroids were washed twice with 1X PBS for an overall time of 20 min and then overlaid by 150 µl CM. The plate was incubated at 37 °C and 5% CO_2_. After 16 h incubation, spheroids were fixed with 4% PFA for further analyses. Sprouting was visualised using an inverted microscope (CellObserver Z1, Zeiss, Germany) and analysed using ZEN software.

In order to deplete ANGPT2 from serum-free CM, a mixture of protein G-coupled Sepharose beads (Santa Cruz, Sc-2002) and protein A-coupled Sepharose beads (Millipore, 92590) with 1:1 ratio was used. Beads were washed with 1X PBS and incubated with ANGPT2 antibody (MyBioSource, MBS2544048) on an orbital shaker with low rotating speed for 1 h at RT. Then, beads were washed from unbound antibodies and incubated with CM (serum-free DMEM) from treated C4-2 cells on the same shaker for 3 h at RT. The immunoprecipitations were pulled down by centrifugation and the immune-depleted CM were used to treat HUVECs spheroids for 16 h.

To inhibit ANGPT2-integrin receptors, the small molecules cilengitide (Selleckchem, S7077) and ATN-161 (Selleckchem, S8454) were used for treating HUVECs spheroids. Spheroids were treated with a final concentration of 10 µM of inhibitors in CM (serum-free) derived from C4-2 cells for 16 h.

### TCGA database analyses

The mapped results (bam file) of RNA sequencing of 456 tumour samples were downloaded from the TCGA (Project#24795) database (gdc.canvcer.gov). To calculate the FPKM (fragments per kilo base pair transcript per million reads), cuffdiff (v2.2.1) was used with --library-norm-method quartile -total-hit-norm parameters with hg38 refseq annotation. The calculated FPKM for each ANGPT2 variant was used for plotting.

### Accession numbers

The RNA-sequencing data is available in the gene expression omnibus (GEO) database under the identifier number: GSE172205. To access use the following password: elspawuervsfzcz

## Supplementary information

Supplement
