# Supplementary material for: The natural compound atraric acid suppresses androgen-regulated neo-angiogenesis of castration-resistant prostate cancer through angiopoietin 2
Source: Oncogene. 2022 May 5;41(23):3263–77. doi: 10.1038/s41388-022-02333-7 (PMC9166678; doi:10.1038/s41388-022-02333-7)
Supplement: Supplementary file 1 — Supplement [file 41388_2022_2333_MOESM1_ESM.pptx]

## Slide 1
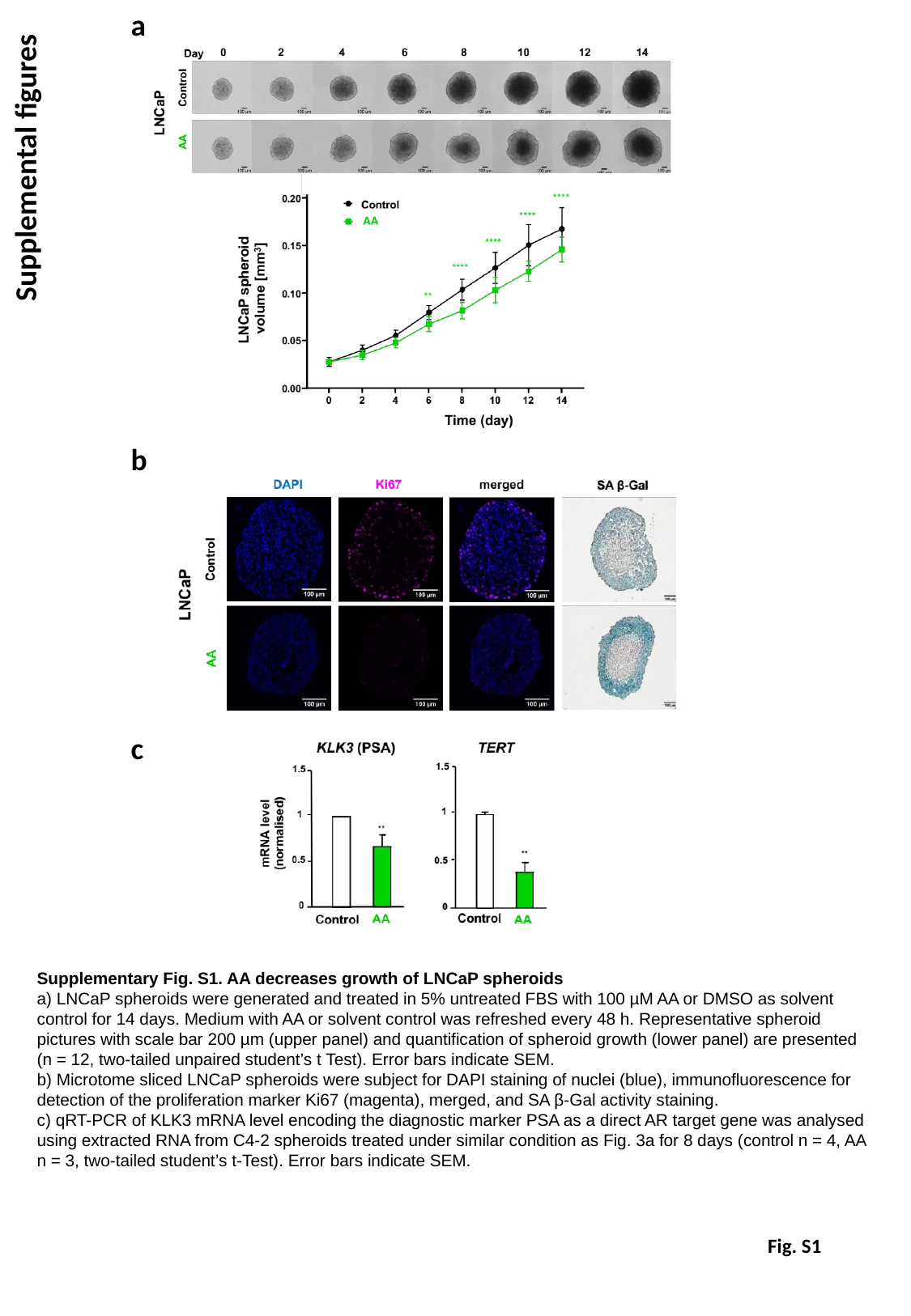

a
Supplemental figures
b
c
Supplementary Fig. S1. AA decreases growth of LNCaP spheroids
a) LNCaP spheroids were generated and treated in 5% untreated FBS with 100 µM AA or DMSO as solvent control for 14 days. Medium with AA or solvent control was refreshed every 48 h. Representative spheroid pictures with scale bar 200 µm (upper panel) and quantification of spheroid growth (lower panel) are presented (n = 12, two-tailed unpaired student’s t Test). Error bars indicate SEM.
b) Microtome sliced LNCaP spheroids were subject for DAPI staining of nuclei (blue), immunofluorescence for detection of the proliferation marker Ki67 (magenta), merged, and SA β-Gal activity staining.
c) qRT-PCR of KLK3 mRNA level encoding the diagnostic marker PSA as a direct AR target gene was analysed using extracted RNA from C4-2 spheroids treated under similar condition as Fig. 3a for 8 days (control n = 4, AA n = 3, two-tailed student’s t-Test). Error bars indicate SEM.
Fig. S1

## Slide 2
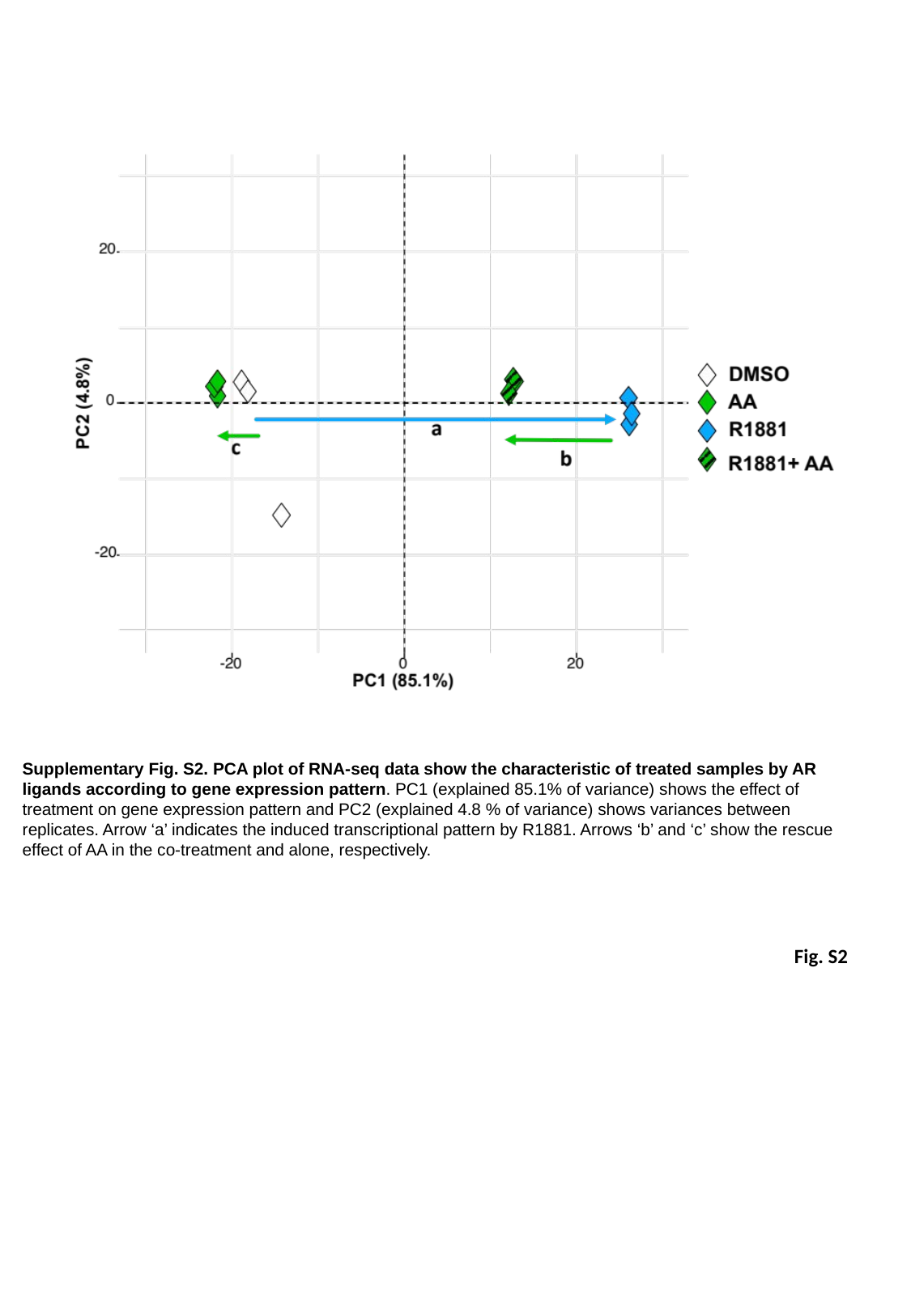

Supplementary Fig. S2. PCA plot of RNA‑seq data show the characteristic of treated samples by AR ligands according to gene expression pattern. PC1 (explained 85.1% of variance) shows the effect of treatment on gene expression pattern and PC2 (explained 4.8 % of variance) shows variances between replicates. Arrow ‘a’ indicates the induced transcriptional pattern by R1881. Arrows ‘b’ and ‘c’ show the rescue effect of AA in the co‑treatment and alone, respectively.
Fig. S2

## Slide 3
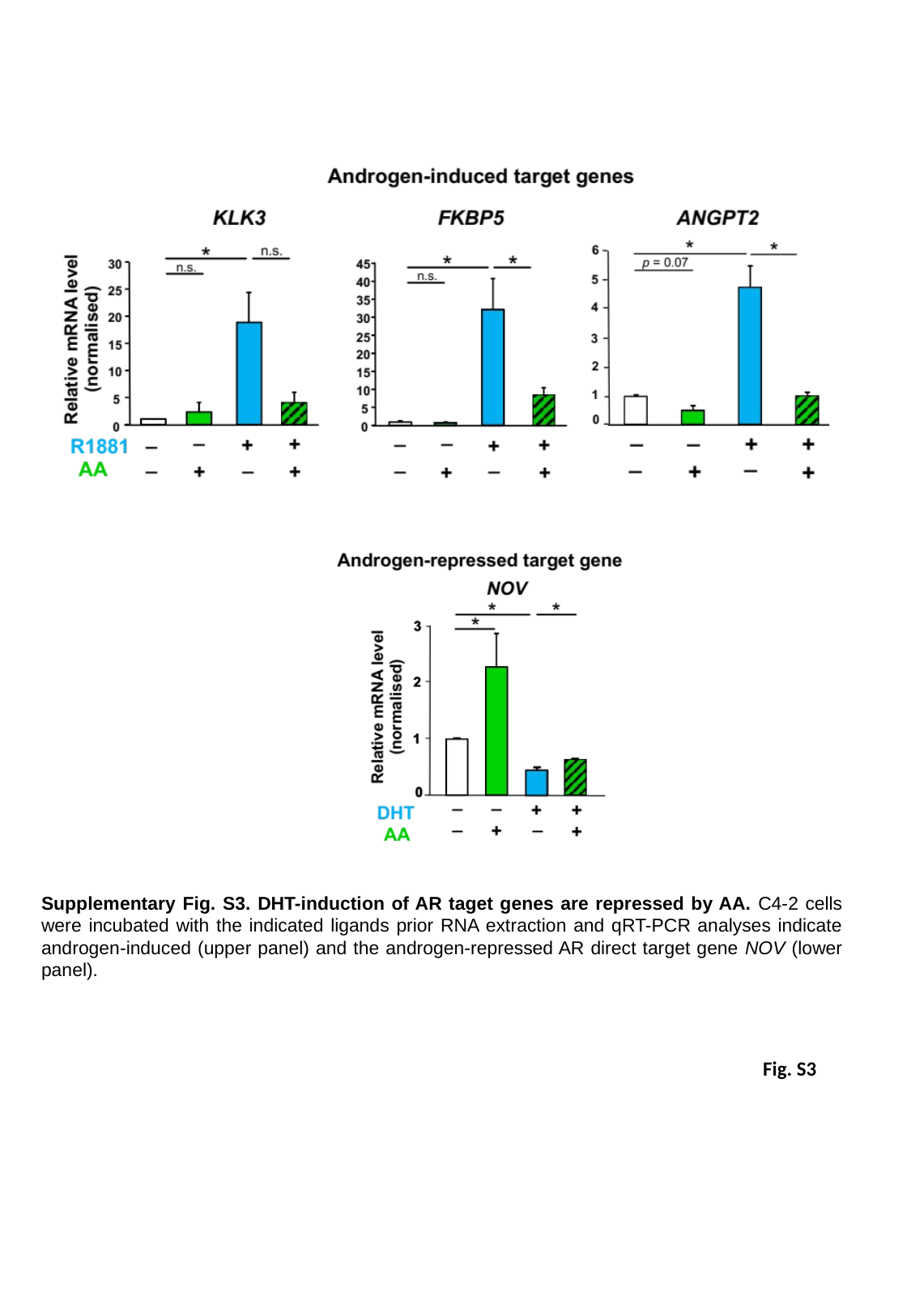

Supplementary Fig. S3. DHT-induction of AR taget genes are repressed by AA. C4-2 cells were incubated with the indicated ligands prior RNA extraction and qRT-PCR analyses indicate androgen-induced (upper panel) and the androgen-repressed AR direct target gene NOV (lower panel).
Fig. S3

## Slide 4
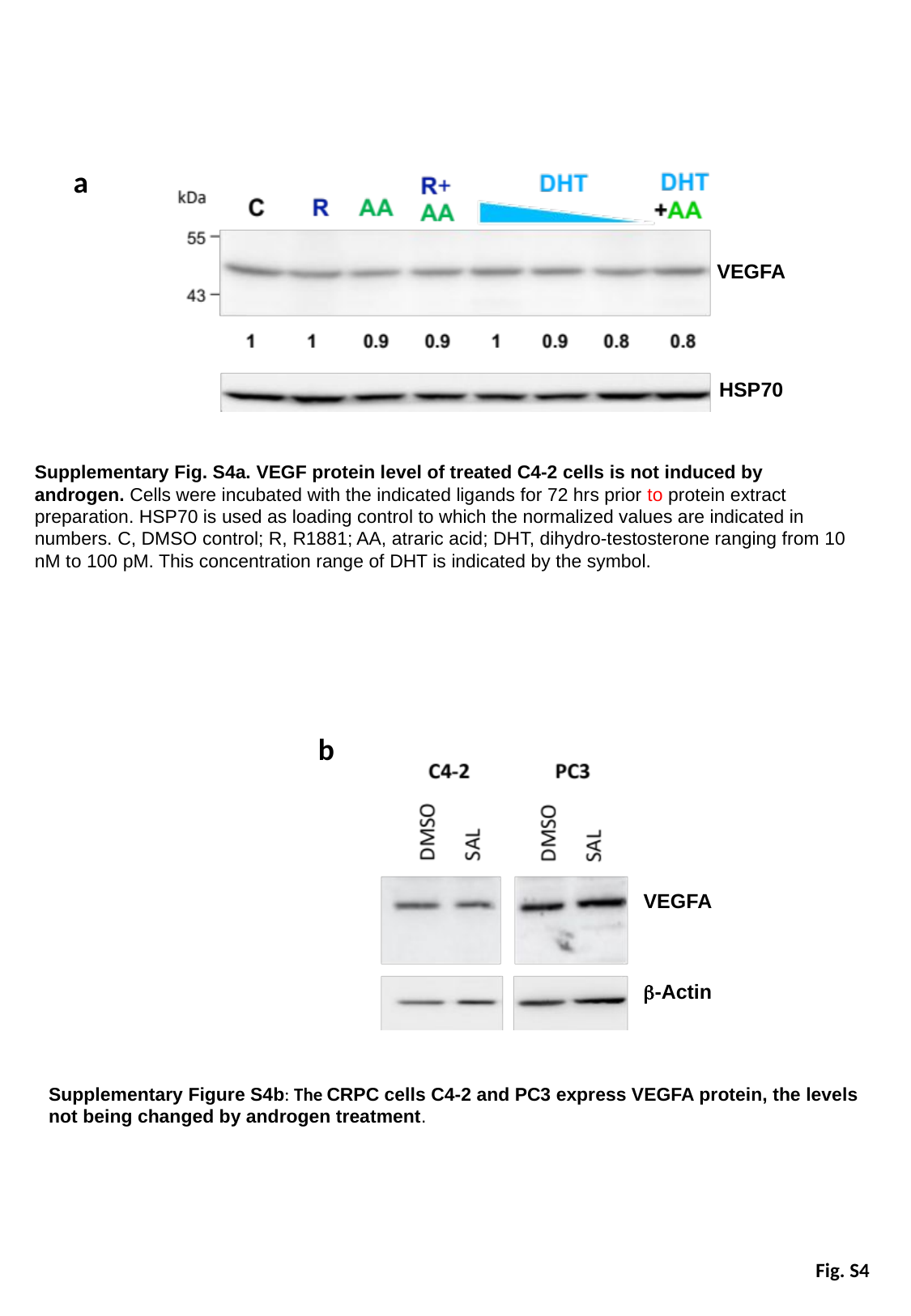

a
VEGFA
HSP70
Supplementary Fig. S4a. VEGF protein level of treated C4-2 cells is not induced by androgen. Cells were incubated with the indicated ligands for 72 hrs prior to protein extract preparation. HSP70 is used as loading control to which the normalized values are indicated in numbers. C, DMSO control; R, R1881; AA, atraric acid; DHT, dihydro-testosterone ranging from 10 nM to 100 pM. This concentration range of DHT is indicated by the symbol.
b
VEGFA
b-Actin
Supplementary Figure S4b: The CRPC cells C4-2 and PC3 express VEGFA protein, the levels not being changed by androgen treatment.
Fig. S4

## Slide 5
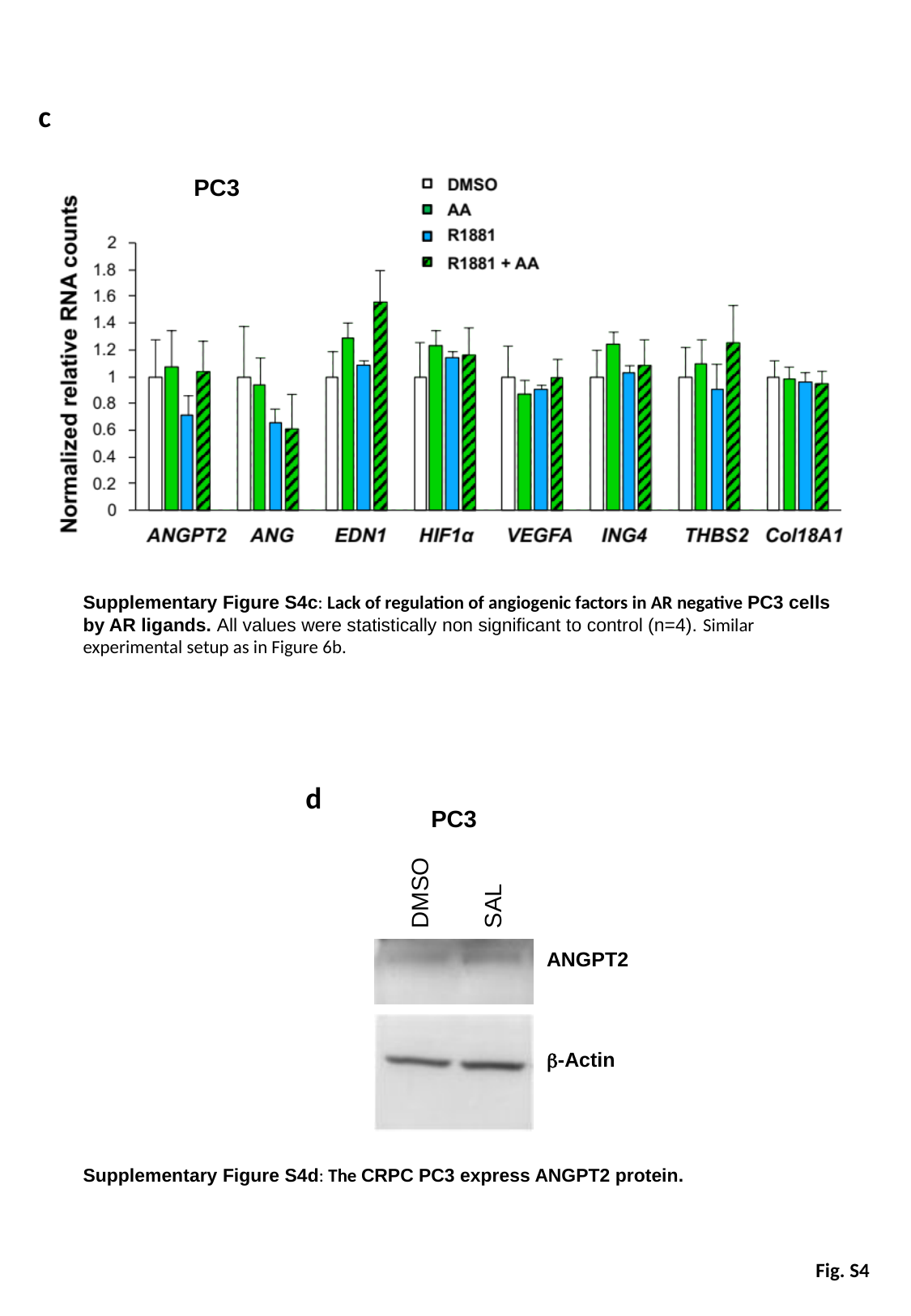

c
PC3
Supplementary Figure S4c: Lack of regulation of angiogenic factors in AR negative PC3 cells by AR ligands. All values were statistically non significant to control (n=4). Similar experimental setup as in Figure 6b.
d
PC3
DMSO
SAL
ANGPT2
b-Actin
Supplementary Figure S4d: The CRPC PC3 express ANGPT2 protein.
Fig. S4

## Slide 6
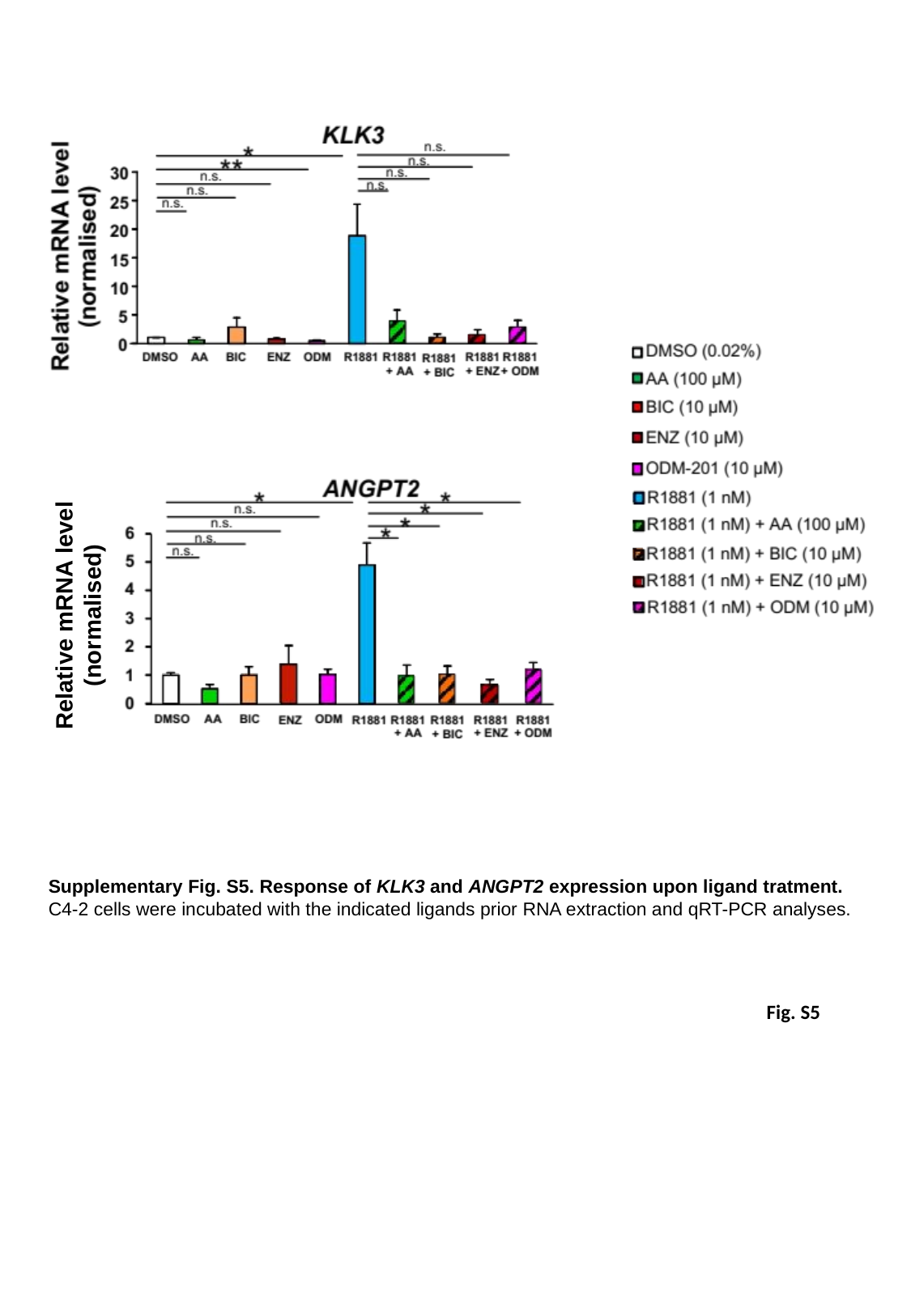

Relative mRNA level (normalised)
Supplementary Fig. S5. Response of KLK3 and ANGPT2 expression upon ligand tratment. C4-2 cells were incubated with the indicated ligands prior RNA extraction and qRT-PCR analyses.
Fig. S5

## Slide 7
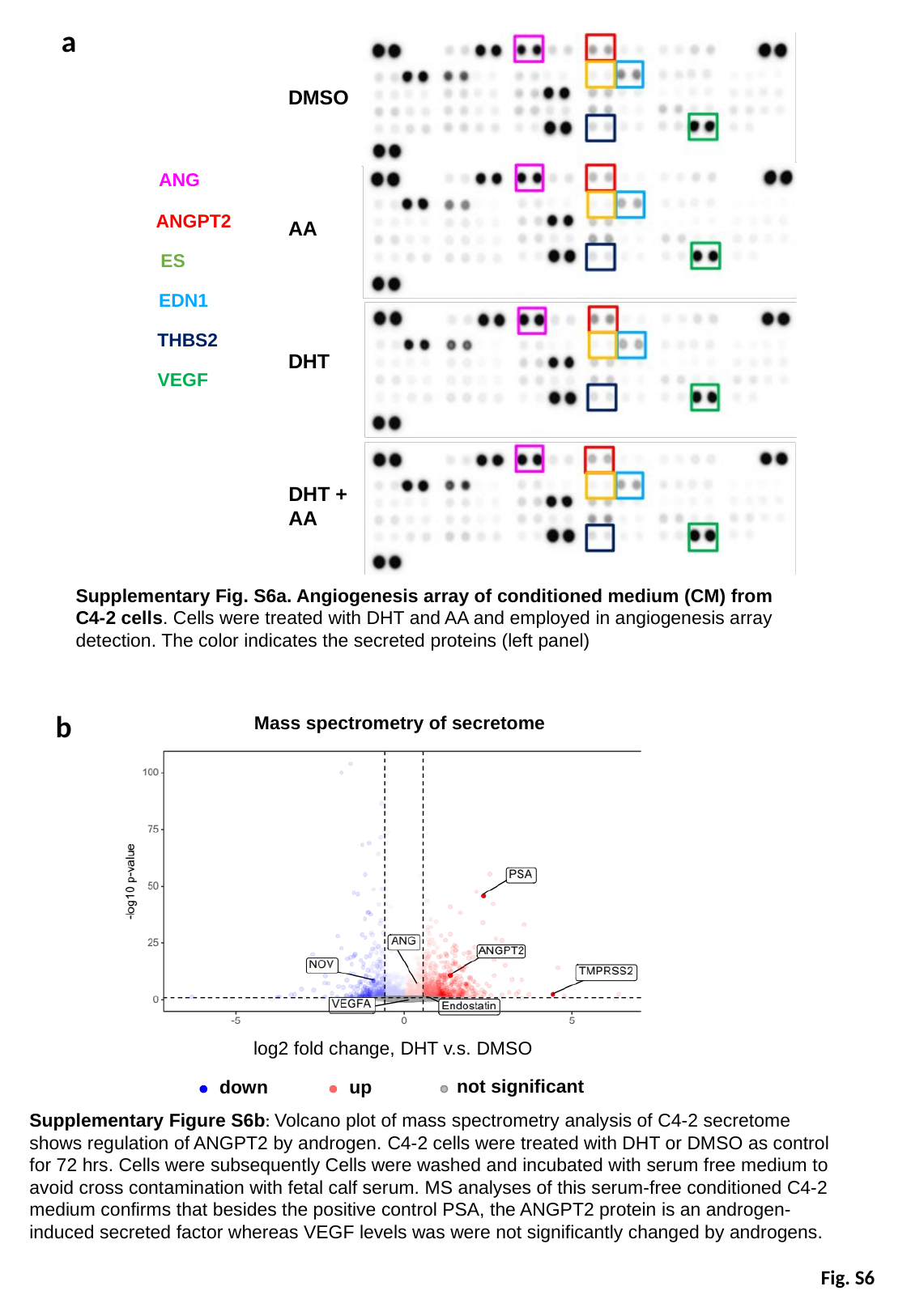

a
DMSO
ANG
ANGPT2
AA
ES
EDN1
THBS2
DHT
VEGF
DHT +
AA
Supplementary Fig. S6a. Angiogenesis array of conditioned medium (CM) from C4-2 cells. Cells were treated with DHT and AA and employed in angiogenesis array detection. The color indicates the secreted proteins (left panel)
b
Mass spectrometry of secretome
log2 fold change, DHT v.s. DMSO
not significant
up
down
Supplementary Figure S6b: Volcano plot of mass spectrometry analysis of C4-2 secretome shows regulation of ANGPT2 by androgen. C4-2 cells were treated with DHT or DMSO as control for 72 hrs. Cells were subsequently Cells were washed and incubated with serum free medium to avoid cross contamination with fetal calf serum. MS analyses of this serum-free conditioned C4-2 medium confirms that besides the positive control PSA, the ANGPT2 protein is an androgen-induced secreted factor whereas VEGF levels was were not significantly changed by androgens.
Fig. S6

## Slide 8
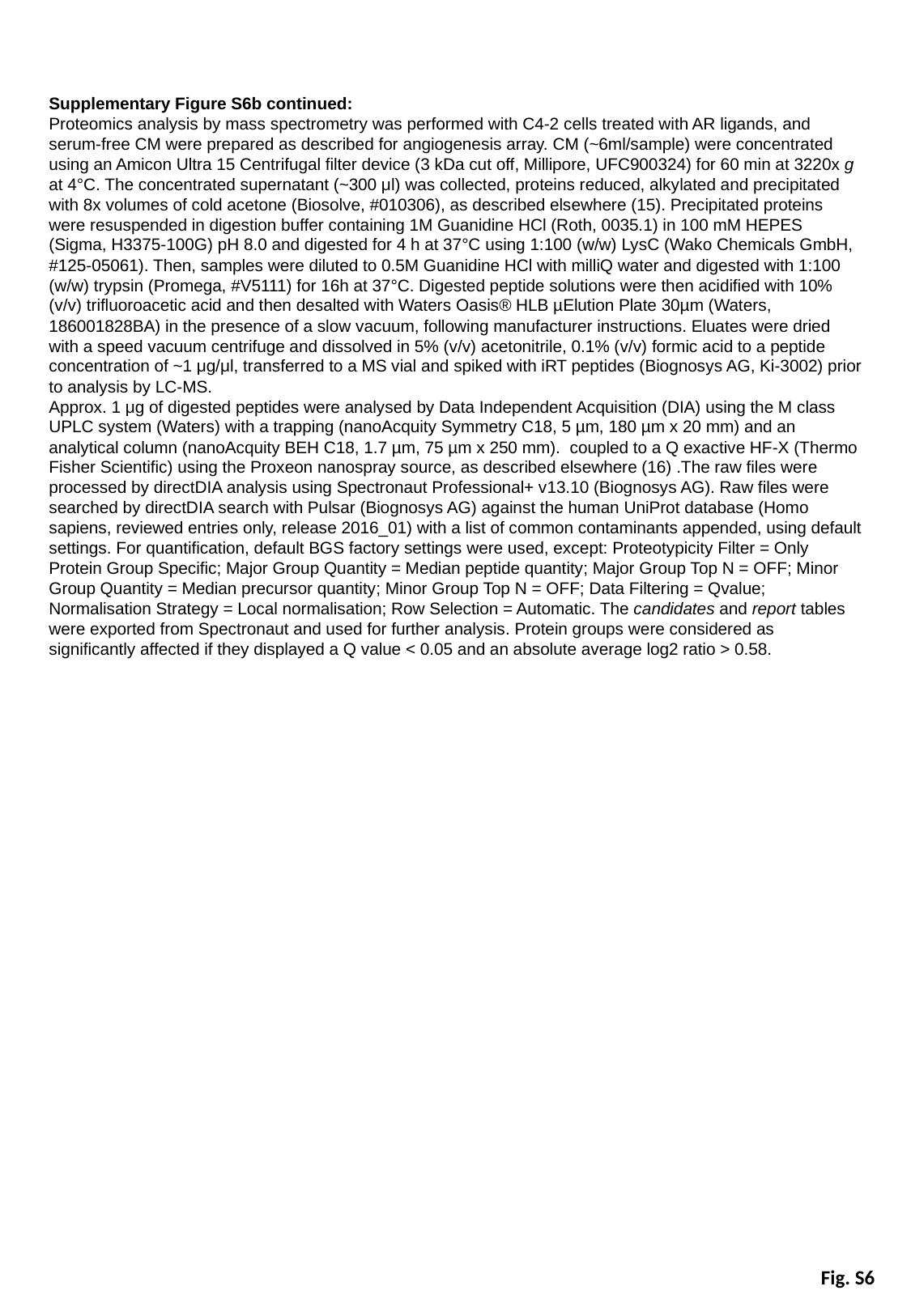

Supplementary Figure S6b continued:
Proteomics analysis by mass spectrometry was performed with C4-2 cells treated with AR ligands, and serum-free CM were prepared as described for angiogenesis array. CM (~6ml/sample) were concentrated using an Amicon Ultra 15 Centrifugal filter device (3 kDa cut off, Millipore, UFC900324) for 60 min at 3220x g at 4°C. The concentrated supernatant (~300 μl) was collected, proteins reduced, alkylated and precipitated with 8x volumes of cold acetone (Biosolve, #010306), as described elsewhere (15). Precipitated proteins were resuspended in digestion buffer containing 1M Guanidine HCl (Roth, 0035.1) in 100 mM HEPES (Sigma, H3375-100G) pH 8.0 and digested for 4 h at 37°C using 1:100 (w/w) LysC (Wako Chemicals GmbH, #125-05061). Then, samples were diluted to 0.5M Guanidine HCl with milliQ water and digested with 1:100 (w/w) trypsin (Promega, #V5111) for 16h at 37°C. Digested peptide solutions were then acidified with 10% (v/v) trifluoroacetic acid and then desalted with Waters Oasis® HLB µElution Plate 30µm (Waters, 186001828BA) in the presence of a slow vacuum, following manufacturer instructions. Eluates were dried with a speed vacuum centrifuge and dissolved in 5% (v/v) acetonitrile, 0.1% (v/v) formic acid to a peptide concentration of ~1 μg/μl, transferred to a MS vial and spiked with iRT peptides (Biognosys AG, Ki-3002) prior to analysis by LC-MS.
Approx. 1 μg of digested peptides were analysed by Data Independent Acquisition (DIA) using the M class UPLC system (Waters) with a trapping (nanoAcquity Symmetry C18, 5 µm, 180 µm x 20 mm) and an analytical column (nanoAcquity BEH C18, 1.7 µm, 75 µm x 250 mm). coupled to a Q exactive HF-X (Thermo Fisher Scientific) using the Proxeon nanospray source, as described elsewhere (16) .The raw files were processed by directDIA analysis using Spectronaut Professional+ v13.10 (Biognosys AG). Raw files were searched by directDIA search with Pulsar (Biognosys AG) against the human UniProt database (Homo sapiens, reviewed entries only, release 2016_01) with a list of common contaminants appended, using default settings. For quantification, default BGS factory settings were used, except: Proteotypicity Filter = Only Protein Group Specific; Major Group Quantity = Median peptide quantity; Major Group Top N = OFF; Minor Group Quantity = Median precursor quantity; Minor Group Top N = OFF; Data Filtering = Qvalue; Normalisation Strategy = Local normalisation; Row Selection = Automatic. The candidates and report tables were exported from Spectronaut and used for further analysis. Protein groups were considered as significantly affected if they displayed a Q value < 0.05 and an absolute average log2 ratio > 0.58.
Fig. S6

## Slide 9
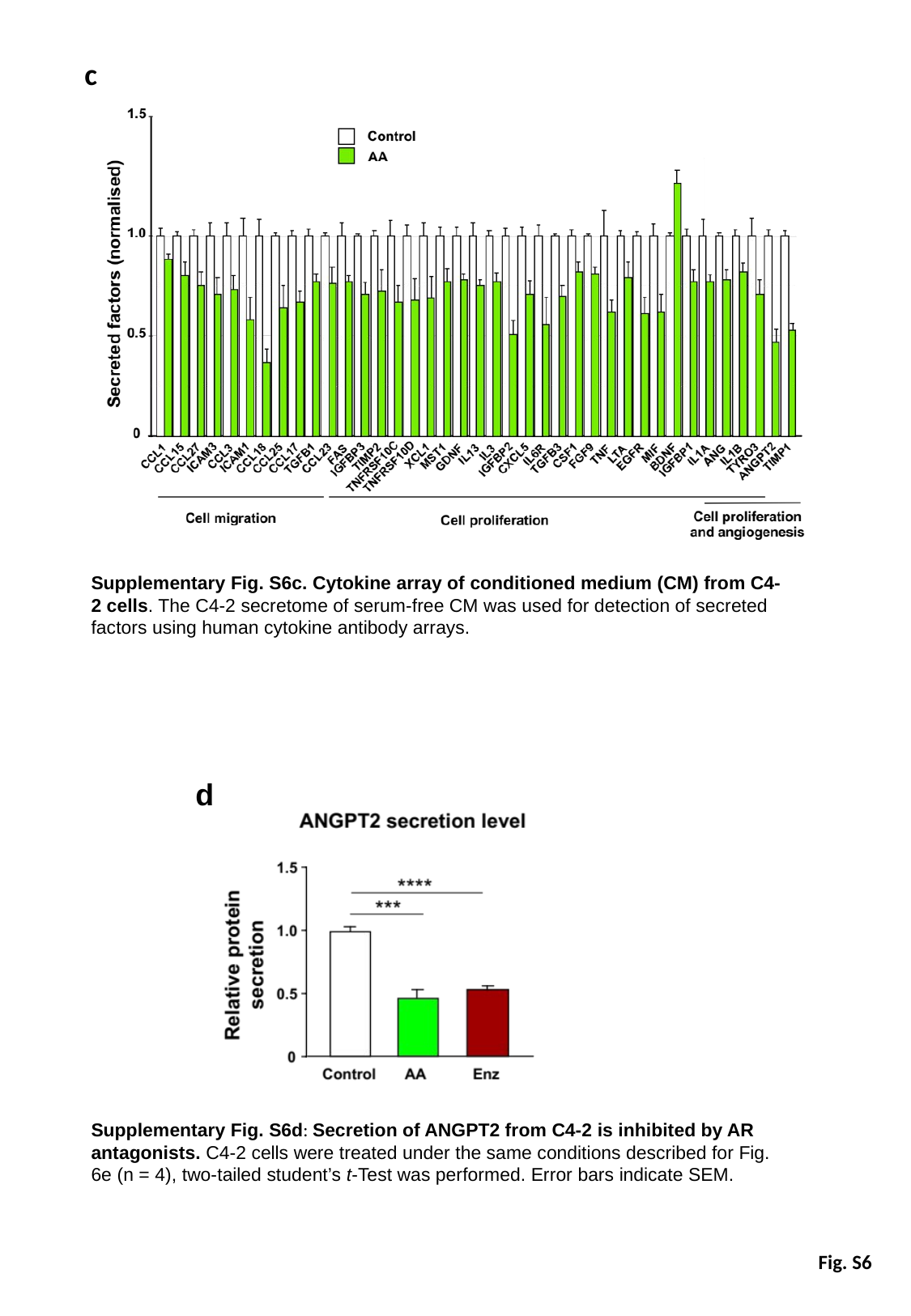

c
Supplementary Fig. S6c. Cytokine array of conditioned medium (CM) from C4-2 cells. The C4-2 secretome of serum-free CM was used for detection of secreted factors using human cytokine antibody arrays.
d
Supplementary Fig. S6d: Secretion of ANGPT2 from C4-2 is inhibited by AR antagonists. C4-2 cells were treated under the same conditions described for Fig. 6e (n = 4), two-tailed student’s t-Test was performed. Error bars indicate SEM.
Fig. S6

## Slide 10
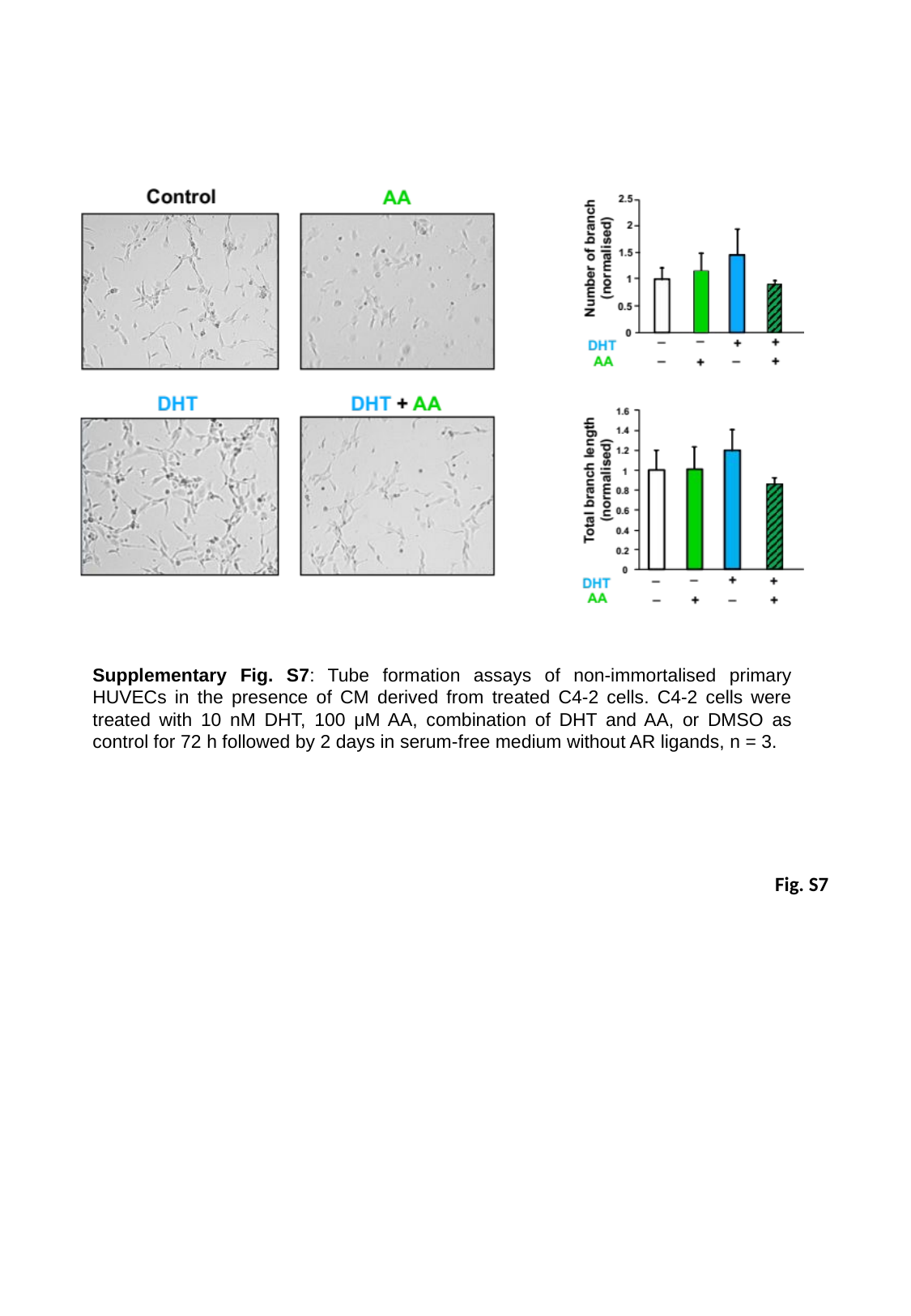

Supplementary Fig. S7: Tube formation assays of non-immortalised primary HUVECs in the presence of CM derived from treated C4-2 cells. C4-2 cells were treated with 10 nM DHT, 100 μM AA, combination of DHT and AA, or DMSO as control for 72 h followed by 2 days in serum-free medium without AR ligands, n = 3.
Fig. S7

## Slide 11
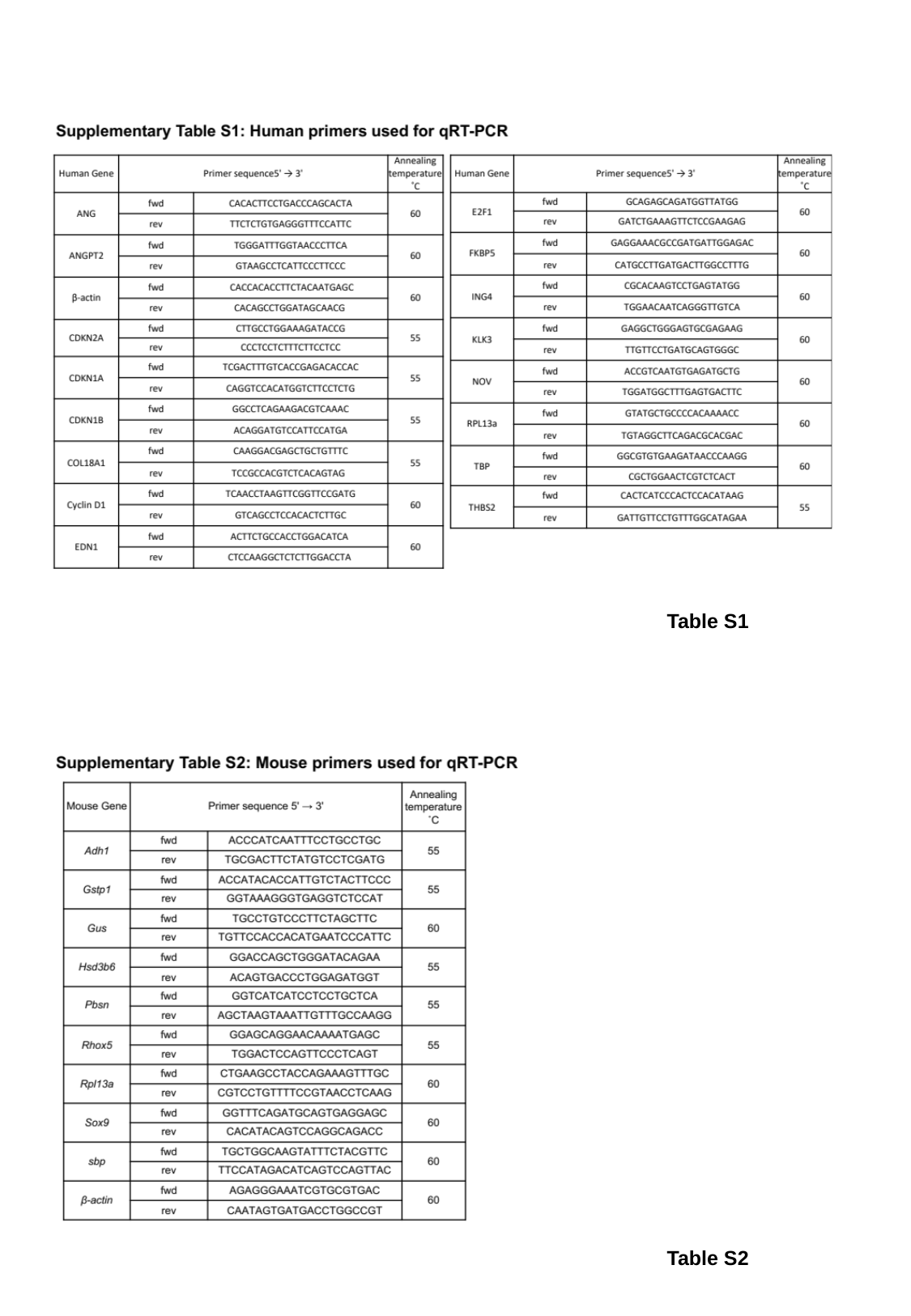

Table S1
Table S2
